# Erbb4 Is Required for Cerebellar Development and Malignant Phenotype of Medulloblastoma

**DOI:** 10.3390/cancers12040997

**Published:** 2020-04-17

**Authors:** Juncal Aldaregia, Peio Errarte, Ane Olazagoitia-Garmendia, Marian Gimeno, Jose Javier Uriz, Timothy R. Gershon, Idoia Garcia, Ander Matheu

**Affiliations:** 1 Cellular Oncology group, Biodonostia Health Research Institute, Dr. Beguiristain s/n, 20014 San Sebastian, Spain; juncal.aldareguia@biodonostia.org (J.A.); peioerrarte@gmail.com (P.E.); aneolaga@gmail.com (A.O.-G.); a123856@alumni.tecnum.es (M.G.); 2 Donostia University Hospital, 20014 San Sebastian, Spain; josejavier.urizmonaut@osakidetza.eus; 3 Department of Neurology, University of North Carolina School of Medicine, Chapel Hill, NC 27516, USA; gershont@neurology.uc.edu; 4 Ikerbasque, Basque Foundation for Science, 48013 Bilbao, Spain; 5 CIBERfes, Carlos III Institute, 28029 Madrid, Spain

**Keywords:** ERBB4, medulloblastoma, cerebellum development, cellular plasticity

## Abstract

Medulloblastoma is the most common and malignant pediatric brain tumor in childhood. It originates from dysregulation of cerebellar development, due to an excessive proliferation of cerebellar granule neuron precursor cells (CGNPs). The underlying molecular mechanisms, except for the role of SHH and WNT pathways, remain largely unknown. ERBB4 is a tyrosine kinase receptor whose activity in cancer is tissue dependent. In this study, we characterized the role of ERBB4 during cerebellum development and medulloblastoma progression paying particular interests to its role in CGNPs and medulloblastoma stem cells (MBSCs). Our results show that ERBB4 is expressed in the CGNPs during cerebellum development where it plays a critical role in migration, apoptosis and differentiation. Similarly, it is enriched in the population of MBSCs, where also controls those critical processes, as well as self-renewal and tumor initiation for medulloblastoma progression. These results are translated to clinical samples where high levels of ERBB4 correlate with poor outcome in Group 4 and all medulloblastomas groups. Transcriptomic analysis identified critical processes and pathways altered in cells with knock-down of *ERBB4*. These results highlight the impact and underlying mechanisms of ERBB4 in critical processes during cerebellum development and medulloblastoma.

## 1. Introduction

Medulloblastoma is the most frequent pediatric solid tumor in childhood [1]. This tumor is defined as a neural progenitor tumor that arises from an abnormal cerebellar development. The complete development of the cerebellum occurs postnatally, and this process needs a well-regulated rate of proliferation and differentiation of the cerebellar granule neuron progenitors (CGNPs) to form the correct structure of the cerebellum [2]. CGNPs proliferate in the external germinal layer (EGL) and migrate along Bergmann radial fibers to the internal granule cell layer (IGL) [3]. When a dysregulation of this process occurs, and CGNPs do not exit the proliferative state, medulloblastoma can be generated [4].

Medulloblastomas are classified by the World Health Organization (WHO) as grade IV lesions [5]. The therapeutic approach used nowadays for medulloblastoma has improved the survival of patients reaching up to 70–90% of survival rate [6]. Despite the improvement in the prognosis for children with medulloblastoma, 30% of surviving patients relapse after the initial treatment [7,8], and the high doses of chemotherapy and radiotherapy used cause permanent neurological sequelae and disability [7,9]. Increasing evidence shows that inter- and intra-tumor heterogeneity play a major role in the aggressiveness and the resistance to the available therapeutic strategies. In this regard, some studies indicate that medulloblastomas are, indeed, a heterogeneous mix of different biological entities [10], and consequently, they have been classified, based on their histopathological features and using molecular characteristics, into 4 different subgroups, namely WNT, SHH, Group 3, and Group 4 [11]. Moreover, intra-tumor cellular heterogeneity has also been experimentally demonstrated, with a population of medulloblastoma stem-like cells (MBSCs) that is characterized by three main properties—quiescence, self-renewal, and differentiation capacity—which are essential for tumor maintenance and growth [12,13]. Remarkably, MBSCs are responsible for drug resistance and the recurrence of the tumor [14]. Together, this information shows the need for new therapeutic approaches in order to reduce the important side effects caused by the current therapy. To achieve this aim, further elucidation of programs governing MBSCs and medulloblastoma progression are needed.

The ERBB family of tyrosine kinase receptors, including EGFR (ERBB1/HER1), ERBB2 (Neu/HER2), ERBB3 (HER3), and ERBB4 (or HER4), has largely been associated with cancer pathogenesis and epithelium-mesenchymal transition (EMT) [15]. Principally, EGFR and HER2 appear mutated in several epithelial tumors and have been implicated in the development and progression of cancer [16]. In contrast, the role of ERBB4 in cancer is more controversial. Thus, high ERBB4 expression has been correlated with shorter overall survival or poor prognosis in glioblastoma [17], lymph node-negative esophageal squamous cell carcinoma [18], ovarian serous carcinoma [19], triple negative breast cancer [20], and colorectal cancer [21]. On the contrary, low expression of ERBB4 protein or mRNA levels have been correlated with shorter overall survival or poor prognosis in hepatocellular carcinoma [22], breast cancer [23], and triple-negative and Her2 positive breast cancer [24]. Moreover, recent studies show the implication of ERBB4 in the development and malignant phenotype progression of colon, lung, ovarian, gastrointestinal, melanoma, and neuroblastoma tumors, among others [25,26]. ERBB4 inhibition also increases tumor cells’ vulnerability prior to chemotherapy treatment in neuroblastoma [27] and in lung cancer [28], but little is known about its role in cerebellar development and medulloblastoma. 

ERBB4 is expressed in the epithelium, mesenchyme, cardiac cells, and neuronal cells [16]. This receptor needs its ligands bound to be activated and regulates different cell processes, such as cell migration, proliferation, survival, and differentiation [29]. ERBB4 plays an important role in the development, ranging from embryogenesis to the development of heart, skin, and central nervous system. Specifically, it is implicated in neuronal migration and pathfinding [30]. Moreover, activated ERBB4 promotes neurogenesis and neuronal differentiation and survival [31,32,33]. It has also been demonstrated that ERBB4 is expressed in CGNPs, and it is involved in cerebellar development [34,35,36]. In fact, ERBB4 is expressed in cerebellum during its development, and this receptor-mediated signaling in the glia appears to be essential for the movement of the CGNPs along the radial glial fibers [37,38]. In medulloblastoma, it has been shown that human tumor samples express higher levels of ERBB4 than the healthy cerebellum, and that this high expression correlates with a poor prognosis [39,40,41]. These results allowed us to hypothesize that ERBB4 might have a key role in cerebellar development and medulloblastoma progression. 

## 2. Materials and Methods

### 2.1. Mouse Models

Conditional ErbB4 (*ErbB4loxP/loxP*) knockout (*KO*) mice were generously shared by Dr Eva Anton (University of North Carolina, UNC Neuroscience Center, Chapel Hill, NC, USA). *ErbB4loxP/loxP* were crossed with *SmoM2loxP/loxP* mice purchased from Jackson Laboratories to produce *Gfap/Math1-Cre/ErbB4+/−/SmoM2* (*SmoM2; ErbB4KO or SmoM2; ErbB4Het*) mice. *Foxn1nu/Foxn1nu* nude mice were also purchased from Jackson Laboratories.

### 2.2. Cell Culture

DAOY and UW228 medulloblastoma cell lines were kindly provided by Dr. Castresana [42], and D283Med, D341Med, CHLA-01-Med, and CHLA-01R-Med cell lines were obtained from the ATCC. DAOY, UW228 and D283Med cells were cultured in DMEM (Gibco, Waltham, MA, USA), supplemented with 10% fetal bovine serum (FBS), L-glutamine, penicillin, and streptomycin (Gibco). D341Med cells were cultured in DMEM, supplemented with 20% FBS, L-glutamine, penicillin, and streptomycin. CHLA-01-Med and CHLA-01R-Med cells were cultured in DMEM/F12 (Gibco) supplemented with B27 (Fisher), L-glutamine, penicillin, streptomycin, and growth factors b-FGF2 and EGF (Sigma). Oncospheres derived from cell lines were cultured in non-treated plates and were grown in DMEM/F12 supplemented with N2 and B27 (Fisher), 40% glucose (Sigma), and growth factors b-FGF2 and EGF for 10 days. Fresh media were added every 3 days. 

### 2.3. CGNP Isolation and Culture

For CGNP isolation, cerebella from p5–7 pups were dissected, dissociated with papain using the Papain Dissociation System (Worthington Biochemical Corporation, Lakewood, NJ, USA) and allowed to adhere to coated culture wells in DMEM/F12 (Life Technologies, Budapest, Hungary) with 25 mmol/L KCl, supplemented with heat-inactivated FBS and N2. After 4 h, media were replaced with identical serum-free media. Where indicated, cells were treated with 0.5 mg/mL SHH (464SH, R&D Systems, Minneapolis, MN, USA), 100 nM HBEGF (4267-10, BioVision, Milpitas, CA, USA), 100 nM *hNRG1* (4730-10, BioVision, Milpitas, CA, USA), and dexamethasone.

### 2.4. Viral Infections

Lentiviral infection was performed as previously described using a multiplicity of infection of 10 for 6 h [43]. For this, pLKO.1 shERBB4 (*sh2* and *sh3*) were obtained from Sigma. Cells transduced with pLKO.1 empty and shRNA plasmids were selected with 2 μg/mL puromycin (Sigma, St. Louis, MO, USA) and maintained with 0.2 μg/mL puromycin.

### 2.5. Tissue Immunofluorescence

Brains were fixed in 4% paraformaldehyde for at least 48 h. Tissue was processed and embedded in paraffin at the UNC Center for Gastrointestinal Biology and Disease Histology core. Sections were deparaffinized, and antigen retrieval was performed using a low-pH citric acid-based buffer. Staining was performed with assistance from the UNC Translational Pathology Laboratory. Slides were scanned using the Leica Biosystems Aperio ImageScope software (12.3.3). The primary antibodies used were anti-phospho-ERBB4 (P-ERBB4, PA5-38501, ThermoFisher, Waltham, MA, USA), anti-HBEGF (1:500, sc-365182, SantaCruz, Dallas, TX, USA), NeuN (MAB377, Millipore, Burlington, MA, USA), GAD1, and GFAP (Z0334, Dako, Santa Clara, CA, USA). Where indicated, nuclei were counterstained with 4′6-diamino-2-phenylindole (DAPI; catalog number D1306; Life Sciences, St. Petersburg, FL, USA).

### 2.6. Cell Immunofluorescence

Immunofluorescence was performed following standard procedures [44]. The primary and secondary antibodies used were anti-phospho-histone H3 (PH3, 1:2000, Ab14955, Abcam, Cambridge, UK), cleaved-caspase-3 (cC3, 1:500, AF835, R&D Systems, Minneapolis, MN, USA), anti-mouse Alexa Fluor 555 IgG (1:500, Invitrogen, Carlsbad, CA, USA), anti-rabbit Alexa Fluor 488 (1:500, Invitrogen, Carlsbad, CA, USA), and anti-rabbit Alexa Fluor 555 (1:500, Invitrogen, Carlsbad, CA, USA). Nuclear DNA staining and the mounting were performed with the Vectashield Hard set Mounting Medium with DAPI counterstain (Vector Laboratories, Burlingame, CA, USA). Pictures were taken in an Eclipse 80i microscope and processed with the NIS Elements Advanced Research Software (Nikon, Tokyo, Japan).

### 2.7. Brain Immunohistochemistry

For immunohistochemistry assay, mouse brain tissue was immunostained as previously described [45] using the primary antibody against NRG1 (MA5-12896, ThermoFisher, Waltham, MA, USA). Stained slides were digitally acquired using an Aperio ScanScope XT (Aperio, Sausalito, CA, USA).

### 2.8. Tumor Immunohistochemistry

Tumors generated in mice were dissected, fixed in 4% paraformaldehyde for 48 h, and embedded in paraffin. Four-micrometer-thick sections were deparaffinized, rehydrated, and heated for 10 minutes in citrate buffer for antigen retrieval. Endogenous peroxidase was blocked with 5% hydrogen peroxide in methanol for 15 min. After incubation with blocking solution, sections were incubated with primary antibodies (KI67, ab15580 (Abcam, Cambridge, UK) and cC3, AF835 (R&D Systems, Minneapolis, MN, USA)) at 37 °C for 2 h. The sections were then washed and incubated with MACH 3 Rabbit Probe HRP-Polymer (M3R531, Biocare Medical, Pacheco, CA, USA). Color was developed with 3,3′Diaminobenzidine (DAB, SPR-DAB-060, Spring Bioscience, Pleasanton, CA, USA) and nuclei were counterstained with hematoxylin. Pictures were taken in an Eclipse 80i microscope and processed with the NIS Elements Advanced Research Software (Nikon, Tokyo, Japan).

### 2.9. RNAscope 

The RNAscope 2.5 Duplex Assay (ACD Bio-systems, Buffalo Grove, IL, USA) was performed according to the ACD protocol for fresh-frozen tissue. ERBB4 (Cat No. 311,801) was used. The probes were amplified according to the manufacturer’s instructions. The target probes were combined with immunohistochemistry (anti-ERBB4) as described above.

### 2.10. Migration Assay

Transwell cell migration was evaluated using 6.5 mm Transwell^®^ chambers with 8.0 μm pore polycarbonate membrane inserts (Corning, #3422). Quantification of migrating cells was performed 48 h after seeding by unstaining the chambers and measuring the absorbance at 570 nm in a MultiSkan Ascent microplate reader (Thermo Scientific, Budapest, Hungary) using the Ascent software.

### 2.11. Cell Viability MTT Assay

In 96-well plates, 500 cells per well for DAOY cells and 5 × 10^3^ cells per well for D283Med cells were seeded in sextuplicate. After 72 h of incubation at 37 °C, 0.5 mg/mL Thiazolyl Blue Tetrazolium Bromide (MTT, Sigma, St. Louis, MO, USA) was added, and after additional 3 h of incubation at 37 °C, the contents of the wells were removed, and formed crystals were resuspended in 150 μL DMSO per well for DAOY cells. However, for D283Med cells, 100 μL of isopropanol + 0.01 M HCl per well were added to the culture media, and the crystals were resuspended by pipetting up and down. In both cell lines, absorbance was measured at 570 nm in a MultiSkan Ascent microplate reader (Thermo Scientific, Budapest, Hungary) using the Ascent software. Cellular viability of the shERBB4 cells was calculated relative to the absorbance of control cells. 

### 2.12. RNA Extraction, Reverse Transcription, and Gene Expression

Total RNA was extracted using Tri Reagent solution (Life Technologies, Budapest, Hungary). Reverse transcription was performed using random primers and the Maxima First Strand cDNA Synthesis Kit (ThermoFisher, Waltham, MA, USA), according to the manufacturer guidelines. For qRT-PCR, 20  ng of cDNA was used to analyze gene expression with KiCqStart SYBR Green (Sigma, St. Louis, MO, USA) in a CFX384 real-time thermal cycler (BioRad, Hercules, CA, USA). Transcript levels were normalized to *GAPDH* and measured using the ΔΔCt relative quantification method.

### 2.13. Western Blot

Immunoblots were performed following standard procedures [45]. Specific antibodies against ERBB4 (4795S, Cell Signaling, Danvers, MA, USA), P-ERBB4 (4757S, Cell Signaling), cC3 (9664S, Cell Signaling), CD133 (ab16518, Abcam, Cambridge, UK), SOX2 (AB5603, Millipore, Burlington, MA, USA), SOX9 (AB5535, Millipore, Burlington, MA, USA), and β-ACTIN (3700S, Cell Signaling, Danvers, MA, USA) were used in this study. For secondary antibodies, horseradish peroxidase (HRP)-linked anti-rabbit (7074S, Cell Signaling, Danvers, MA, USA) or anti-mouse (7076S, Cell Signaling, Danvers, MA, USA) were used. Detection was performed by chemiluminescence using SuperSignal West Femto Maximun Sensitive Substrate (#34096, ThermoFisher, Waltham, MA, USA).

### 2.14. Colony Formation Assay

The infected cells were seeded into 6-well plates at a density of 500 cells/well. After approximately 15 days from plating, the colonies were fixed with 37% paraformaldehyde and stained with 5% Giemsa. Cell colonies were counted and *shERBB4* colony formation capacity was calculated relative to a colony’s number of control cells.

### 2.15. Oncosphere Formation Assay

To perform the oncospheres assay, 10 × 10^3^ cells were plated in non-treated 6- or 12-well flat bottom plates, for DAOY and D283Med cells respectively, in triplicate, growing them in DMEM/F12 complete medium. Fresh medium was added every 3 days. After 10 days, primary (1ry) oncospheres were counted. Then, spheres were disaggregated with Accutase (Gibco, Waltham, MA, USA), seeded for secondary (2ry) oncospheres, and maintained for another 10 days in culture. 

### 2.16. Cell Cycle

For the cell cycle assay, cells were fixed with 70% ethanol and incubated with RNase A and TO-PRO-3 (Invitrogen, Waltham, MA, USA). Then, cell cycle assay was performed and analyzed by Inbiomed Flow Cytometry facility.

### 2.17. Cell Apoptosis Assay

For Annexin-V determination, Annexin-V Alexa Fluor 488 conjugate apoptosis detection kit (A13201, ThermoFisher, Waltham, MA, USA) was used to measure cell apoptosis, according to the manufacturer’s instructions. Apoptotic rates were subsequently determined using a flow cytometer (Beckman Coulter Gallios, Indianapolis, IN, USA).

### 2.18. In Vivo Carcinogenesis

All animal handling and protocols were approved by the animal care ethics committee of Biodonostia Health Research Institute (CEEA17/016). For subcutaneous injection, DAOY and D283Med cells were harvested with trypsin/EDTA and resuspended in PBS. Approximately 1 × 10^6^, 1 × 10^5^, and 5 × 10^4^ DAOY cells and 1 × 10^6^ and 1 × 10^5^ D283Med cells were injected subcutaneously into both flanks of 8-week-old Foxn1nu/Foxn1nu nude mice. Mice were observed on a weekly basis and external calipers were used to measure tumor size. From these measurements, tumor volume was estimated by V = L × W^2^ × 0.5; where L is the tumor length and W is the tumor width.

### 2.19. Microarray Analysis

Clariom S Human (ThermoFisher, Waltham, MA, USA) microarrays were performed with *ERBB4* knocked-down DAOY cells by the Biodonostia Genomics Facility. Results were analyzed using Transcriptome Analysis Console (TAC, ThermoFisher, Waltham, MA, USA) and GSEA software (Broad Institute, Cambridge, MA, USA). 

### 2.20. Data Analysis

Results are represented as mean values ± standard error (SEM). Statistical significance was calculated using Student’s *t*-test, (* *p* ≤ 0.05; ** *p* ≤ 0.01; and *** *p* ≤ 0.001). Additional tests are included in the text.

## 3. Results

### 3.1. ERBB4 is Expressed in The Inner Part of the External Germinal Layer During Cerebellum Development

**Figure 1:**
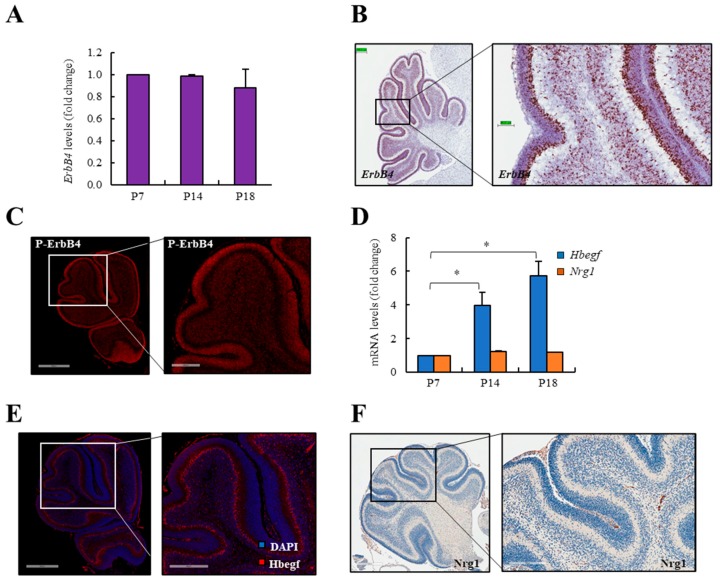
ERBB4 is expressed in the external germinal layer during cerebellum development. (**A**) *ErbB4* mRNA expression in mice cerebellum at postnatal day 7 (p7), p14, and p18. (**B**) Representative RNAscope images of *ErbB4* mRNA expression in p10 mouse cerebellum (*n* ≥ 3). Scale bars: left = 199.8 µm and right = 49.91 µm. (**C**) Representative immunofluorescence images of P-ERBB4 protein expression in p7 mouse cerebellum (*n* ≥ 3). Scale bars: left = 600 µm and right = 200 µm. (**D**) *Hbegf* and *Nrg1* mRNA expression in mice cerebellum at p7, p14, and p18. (**E**) Representative immunofluorescence images of HBEGF protein expression in p7 mouse cerebellum (*n* ≥ 3). Scale bars: left = 500 µm and right = 300 µm. (**F**) Representative immunohistochemistry images of NRG1 protein expression in p7 mouse cerebellum. Scale bars: left = 400 µm and right = 200 µm. * *p* ≤ 0.05.

To investigate the expression of ERBB4 in the cerebellum, we first measured its mRNA levels in the whole developing cerebellum at different time points (postnatal day 7 (p7), p14, and p18). *ErbB4* levels were similar at the different stages within the cerebellum development (Figure 1A). Next, we performed an RNAscope analysis and immunofluorescence assays in p7. These experiments revealed that *ErbB4* is expressed in the inner part of the EGL (Figure 1B), whereas the active form of ERBB4 (P-ERBB4) is expressed in the whole EGL (Figure 1C). The EGL is the proliferative layer of the developing cerebellum. The external part of this layer is very proliferative, whilst the inner part is the more differentiated side of the EGL. ERBB4 can be activated by different ligands. Among them, HBEGF, which is expressed in developing cerebellum by Purkinje cells and it seems to be the dominant ligand in this brain structure [46], and NRG1, which has been observed in the cerebellum EGL [47], are within the most relevant ones. The expression of both ligands was measured at p7, p14, and p18 stages of cerebellum development. The expression of *Hbegf* increased from p7 to p18, whilst *Nrg1* was maintained constant (Figure 1D). Moreover, their expression was assessed by immunofluorescence and immunohistochemistry assays, respectively. HBEGF is expressed in the outer part of the IGL (Figure 1E), where cells differentiated to neurons are located. However, NRG1 expression was very low or undetectable (Figure 1F), together revealing their different pattern of expression during cerebellar development in vivo. 

### 3.2. ERBB4 Is Expressed in Culture CGNPs, and Its Activation Protects Cells from an Apoptotic Stimulus In Vitro

**Figure 2:**
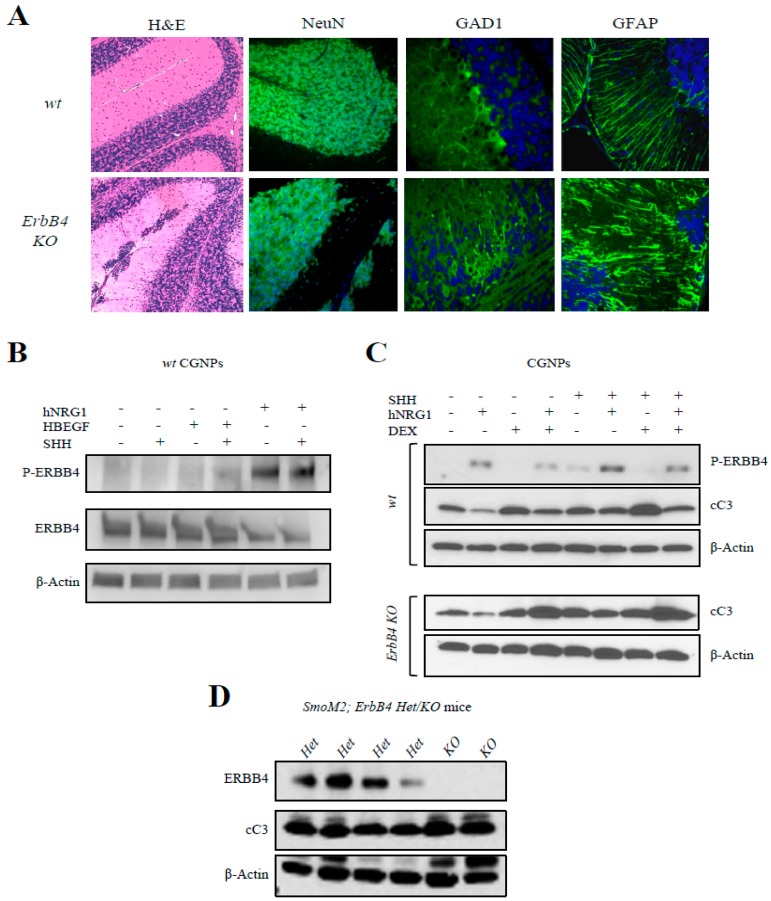
ERBB4 deletion impairs migration during cerebellum development and its activation protects CGNPs from apoptosis. (**A**) Representative immunofluorescence images of the indicated proteins in p20 *wt* and *ErbB4 KO* mouse cerebellum. (**B**) Western blot analysis of indicated proteins in CGNPs from *wt* p5 mice treated with the indicated compounds (*n* ≥ 3). (**C**) Western blot analysis of the indicated proteins in CGNPs from *wt* and *ErbB4 KO* p5 mice treated with the indicated compounds (*n* ≥ 3). (**D**) Western blot analysis of the indicated proteins obtained from p15 cerebellum from *SmoM2; ErbB4 Het* or *KO* mice. The uncropped blots and molecular weight markers are shown in Figures S8–S10.

To assess the role of ERBB4 in CGNPs, we took advantage of *ErbB4* mutant mice that lack ERBB4 expression in Math1 expressing cells, a specific marker of CGNP cells [48]. We analyzed the cerebellum of p20 in control and *ErbB4 KO* mice. At this time point, all the CGNPs should have migrated to the IGL. However, the Hematoxylin and Eosin staining images showed that these cells were still located in the EGL in the *ErbB4* mutant mice (Figure 2A). These results confirm that ERBB4 plays a role in CGNPs migration. We could detect NeuN positive staining in the cells remaining in the EGL in the mutant mice suggesting that the neuronal differentiation process of the CGNPs was not disrupted by the lack of *ErbB4* (Figure 2A). On the contrary, immunofluorescence showed disorganization in the Purkinje cells in *ErbB4 KO* mice (Figure 2A), visualized by the Purkinje cell GAD1 marker [49], since they did not form the straight layer we observed in the *wt* images. Moreover, *ErbB4* mutant mice also displayed disorganization of the astrocytes, based on the GFAP marker (Figure 2A). All these results demonstrate that ERBB4 has an essential role in the organization and normal development of the cerebellum and that its deficit is causing aberrant migration and differentiation of the CGNPs.

To further investigate the role of ERBB4 activation in this population, CGNPs from p5 mice cerebella were isolated and cultured in vitro. Cells were treated with exogenous SHH in order to maintain a proliferative culture and with exogenous HBEGF and human NRG1 (hNRG1) in order to activate the ERBB4 receptor. In these experiments, when treating with exogenous HBEGF, ERBB4 activation was low (Figure 2B), whereas when treating the cells with exogenous hNRG1, a strong activation of ERBB4 could be observed, as there was an increase in P-ERBB4 levels (Figure 2B). These results show different response of ERBB4 to both ligands in vivo and in vitro.

Then cells from *wt* and *ErbB4* mutant mice were cultured and isolated CGNPs were treated again with exogenous SHH, hNRG1, and dexamethasone (a cell apoptosis activator). The results obtained in *wt* mouse cultures showed that when ERBB4 is activated with hNRG1, cells are less sensible to dexamethasone apoptosis activation since cleaved-Caspase-3 (cC3) expression levels are lower in this condition (Figure 2C). However, when the same experiments were repeated with *ErbB4 KO* mice cultured cells, we saw augmented cC3 levels even when cells were treated with exogenous hNRG1 (Figure 2C). This demonstrates that ErbB4 receptor is responsible of apoptotic protection in CGNP cells. 

Next, we crossed *ErbB4* mutant mice with *hGFAPcre;SmoM2* mice, which develop medulloblastoma spontaneously within 15–17 days after birth, and compared ErbB4 *Het* and *KO* mice. Results showed an increase in cC3 levels in samples from *KO* mice compared to *Het* (Figure 2D, and Figure S1). This demonstrates that ERBB4 confers resistance to apoptosis in CGNPs in vitro and in vivo, proposing the inhibition of ERBB4 as a possible therapeutic approach activating apoptosis.

### 3.3. ERBB4 Expression Is Higher in Group 4 Medulloblastomas, and Its High Expression Is Associated with Poor Clinical Outcome

**Figure 3:**
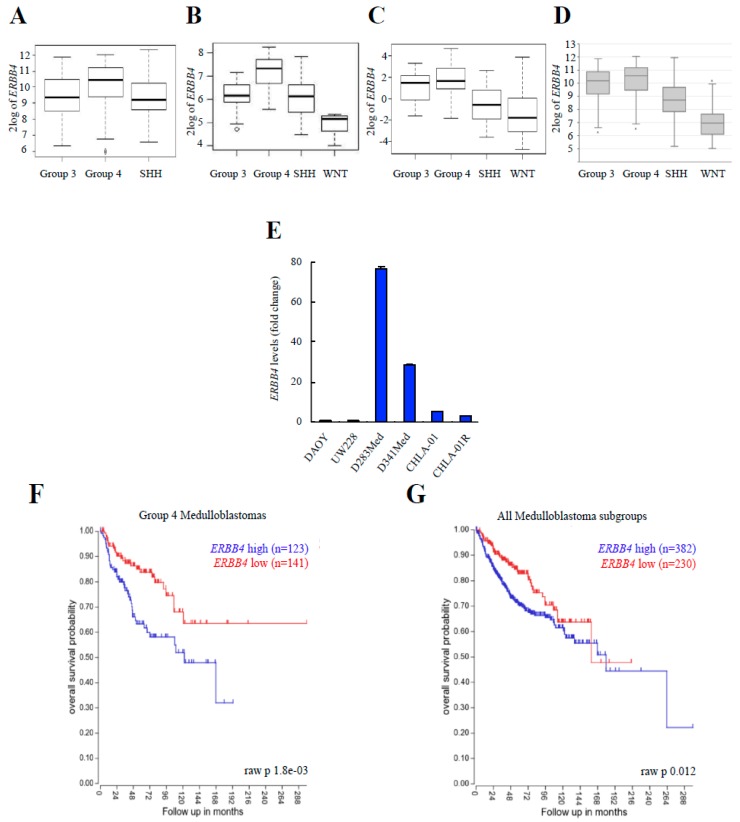
High levels of *ERBB4* are found in Group 4 medulloblastomas and are associated with poor clinical outcome. (**A**–**D**) Boxplot of the log2 of *ERBB4* expression in the indicated medulloblastoma subgroups in (**A**) Northcott et al. (2012) cohort (*n* = 1087), (**B**) Robinson et al. (2012) cohort (*n* = 76), (**C**) Remke et al. (2011) cohort (*n* = 64), and (**D**) Cavalli et al. (2017) cohort (*n* = 612). (**E**) *ERBB4* mRNA expression in the indicated medulloblastoma cell lines (*n* ≥ 3). (**F**,**G**) Kaplan–Meier curves for the Cavalli et al. (2017) cohort’s Group 4 patients’ and all patients’ overall survival rates based on *ERBB4* expression obtained from hgserver1.

Next, we investigated the impact of ERBB4 in human medulloblastoma progression. For this, we analyzed the expression of ERBB4 in different databases of human clinical biopsies from medulloblastoma tumors. First, we compared the expression of ERBB4 in the four different established medulloblastoma groups. We found that Group 4 medulloblastomas presented the highest expression of ERBB4 in all the databases analyzed (Figure 3A–D, and Figure S2A–D). We also studied the expression of ERBB4 in several human medulloblastoma cell lines and detected higher levels in cells belonging to Group 4 (D283Med, D341Med, CHLA-01, and CHLA-01R) compared to DAOY and UW228 from the SHH subgroup (Figure 3E). Furthermore, we investigated the correlation of clinical characteristics with the expression levels of ERBB4 in publicly available datasets of the Cavalli cohort. Strikingly, high ERBB4 expression was associated with shorter overall survival in Group 4 medulloblastomas (*p* = 1.8 × 10^−3^; Figure 3F). Moreover, high ERBB4 expression was also associated with shorter overall survival in the whole cohort (*p* = 0.012; Figure 3G). Altogether, these results show that ERBB4 expression correlates with poor prognosis in all medulloblastomas groups, but it is especially elevated in Group 4 samples, where its high levels are a prognostic biomarker.

### 3.4. ERBB4 Knock-down Inhibits Cell Viability and Activates Apoptosis in Human Medulloblastoma Cells In Vitro and In Vivo

**Figure 4:**
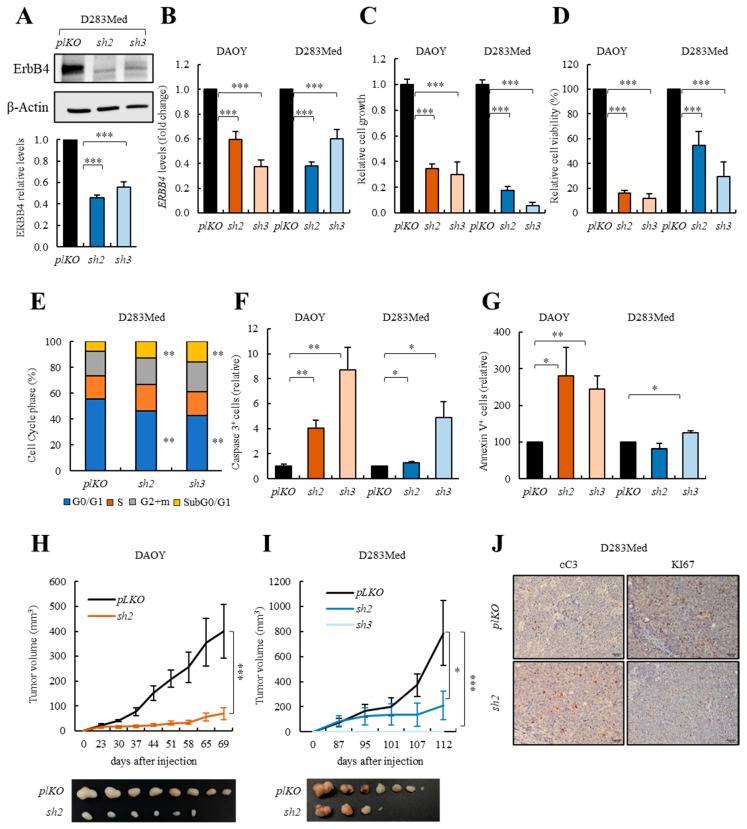
*ERBB4* knock-down impairs medulloblastoma cell viability in vitro and tumor progression in vivo. (**A**) Representative image and quantification of Western blot analysis of ERBB4 in control (*pLKO)* and *shERBB4* (*sh2* and *sh3*) D283Med cells (*n* = 4). (**B**) *ERBB4* mRNA expression in control (*pLKO*) and *shERBB4* (*sh2* and *sh3*) cells (*n* ≥ 9). (**C**) Relative cell growth at day 5 comparing *pLKO* with *sh2* and *sh3* cells (*n* ≥ 6). (**D**) MTT studies measuring cell viability in *sh2* and *sh3* relative to *pLKO* cells (*n* ≥ 6). (**E**) Cell cycle assay measuring the number of cells in each cell cycle phase in *pLKO, sh2*, and *sh3* D283Med cells (*n* ≥ 3). (**F**) Immunofluorescence quantification of cleaved-Caspase-3 (cC3) positive cells in *sh2* and *sh3* relative to *pLKO* DAOY and D283Med cells (*n* ≥ 4). (**G**) Percentage of Annexin-V positive cells in *pLKO, sh2* and *sh3* DAOY and D283Med cells (*n* ≥ 3). (**H**,**I**) Volume of tumors generated after subcutaneous injection of DAOY and D283Med *pLKO, sh2* and *sh3* cells (*n* ≥ 8) at the indicated time points. (**J**) Representative images of the immunohistochemical staining of cC3 and KI67 in tumors generated after subcutaneous injection of D283Med *pLKO* and *sh2* cells (*n* = 5). Scale bars = 100 µm. The uncropped blots and molecular weight markers are shown in Figure S11. * *p* ≤ 0.05; ** *p* ≤ 0.01; and *** *p* ≤ 0.001.

We investigated if ERBB4 plays a role in medulloblastoma progression. To achieve this aim, we knocked-down *ERBB4* expression in DAOY and D283Med cells with two independent shRNAs. Western blot and qRT-PCR revealed inhibition of *ERBB4* when using both *shERBB4* constructs (*sh2* and *sh3*) in DAOY and D283Med cells (Figure 4A,B). Functionally, *ERBB4* silencing promoted a significant decrease of more than 2-fold in cell growth rates (Figure 4C and Figure S3A). In line with this, MTT studies showed that the cell viability rate was also diminished in *ERBB4* silenced DAOY and D283Med cells (Figure 4D). Nevertheless, these phenotypes did not seem to correlate with a reduction in proliferation, since the number of the proliferative marker phospho-histone H3 (PHH3) was similar between both conditions in DAOY cells (Figure S3B). To further characterize the origin of the decreased cell viability, we performed a cell cycle assay. This experiment showed an increase in subG0/G1 in *ERBB4* knock-down cells (Figure 4E and Figure S3C), suggesting an increase in cell death when knocking-down *ERBB4*. We confirmed this increase by quantifying the levels of both cC3 and Annexin-V apoptotic markers, which were elevated in DAOY and D283Med *sh2* and *sh3* cells (Figure 4F,G and Figure S3D). Finally, we observed that *ERBB4* knock-down resulted in a significant decrease in the migratory potential of medulloblastoma cells (Figure S3E). These results further highlight the relevance of ERBB4 in maintaining critical cellular phenotypes, mainly apoptosis and migration, during homeostasis and pathological conditions. 

Next, we determined whether ERBB4 could regulate tumor growth in vivo and injected *sh2* and *sh3* DAOY and D283Med cells subcutaneously in immunocompromised mice. Interestingly, there was a significant decrease of over 70% in tumor growth in *shERBB4* DAOY and D283Med cells, especially with *sh3* that did not form tumors in D283Med cells (Figure 4H,I). Validating these results, weight of tumors generated from knocked-down cells was smaller than empty vector controls (Figure 4H,I and Figure S4A). Moreover, immunohistochemistry revealed increased apoptosis (higher cC3 positive cells) and decreased proliferation (lower KI67 positive cells) in *sh2* DAOY and D283Med cells (Figure 4J and Figure S4B). Altogether, these results showed that *ERBB4* is required for medulloblastoma cell survival and hence tumor progression.

### 3.5. MBSCs Express High Levels of ERBB4 and its Knock-Down Inhibits MBSCs Activity

**Figure 5:**
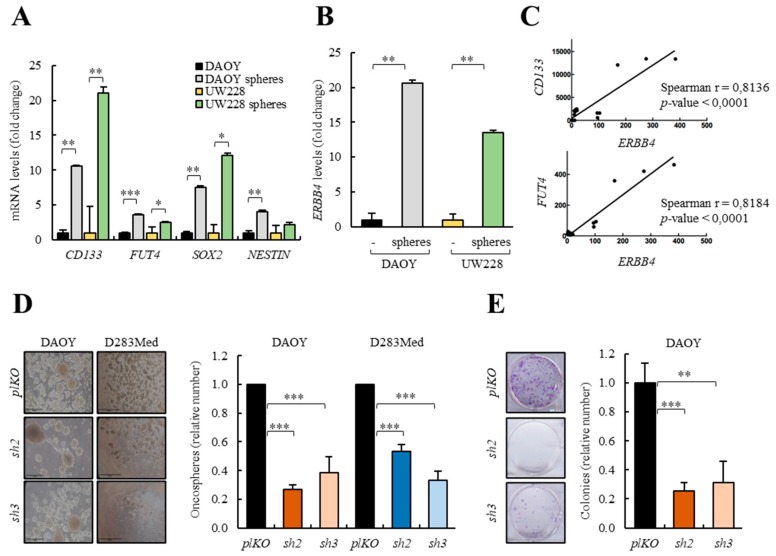

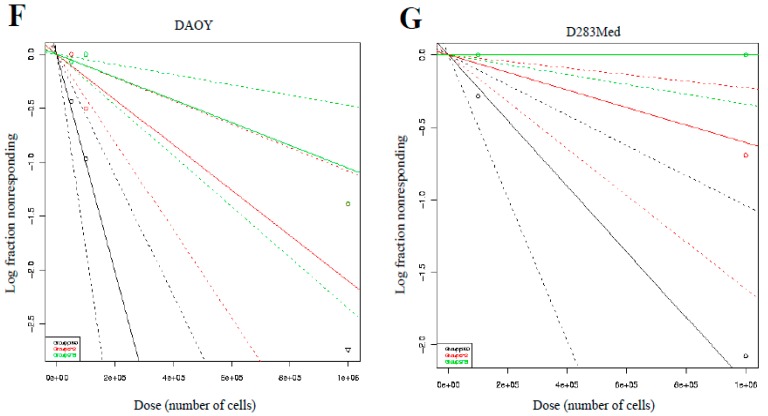
*ERBB4* expression regulates medulloblastoma stem cell (MBSC) activity. (**A**) mRNA levels of the indicated stem cell markers of DAOY and UW228 cells grown in serum or in stem cell media as spheres (*n* ≥ 3). (**B**) *ERBB4* mRNA expression levels of DAOY and UW228 cells grown in serum and stem cell media (*n* ≥ 3). (**C**) Correlation between *CD133* and *FUT4* expression levels and *ERBB4* expression levels in different medulloblastoma cell lines (DAOY, UW228, D283Med, D341Med, CHLA-01-Med, and CHLA-01R-Med) (*n* ≥ 3). (**D**) Representative images and quantification of the number of oncospheres formed from the indicated conditions in DAOY and D283Med cells (*n* ≥ 4). Scale bars = 100 µm. (**E**) Representative images and quantification of the number of colonies formed from the indicated conditions in DAOY cells (*n* ≥ 3). (**F**) Frequency of tumor initiation after subcutaneous injection in nude mice of 1 × 10^6^, 1 × 10^5^ and 5 × 10^4^ DAOY cells transduced with *pLKO*, *sh2* and *sh3*. (**G**) Frequency of tumor initiation after subcutaneous injection in nude mice of 1 × 10^6^ and 1 × 10^5^ D283Med cells transduced with *pLKO*, *sh2* and *sh3*. The incidence of tumor initiation was measured using the ELDA platform (black line represents *pLKO* condition, red line represents *sh2*, and green line represents *sh3*). * *p* ≤ 0.05; ** *p* ≤ 0.01; and *** *p* ≤ 0.001.

In order to investigate the role of ERBB4 in MBSCs, we cultured two conventional DAOY and UW228 medulloblastoma cell lines, with low endogenous levels of *ERBB4*, and D283Med and D341Med, with high expression of the receptor, in the presence of serum and also in stem cell media to form oncospheres. We verified that the stem culture condition increased the expression of well-established MBSC markers *CD133*, *FUT4*, *SOX2, NESTIN* [12,50,51] in all cell lines compared to the expression in medulloblastoma cells cultured in the presence of serum (Figure 5A and Figure S5A,B). Of note, oncospheres from DAOY and UW228 cells expressed higher levels of *ERBB4* than cells cultured in the presence of serum (Figure 5B and Figure S5B). In addition, we performed a correlation analysis of the expression of *ERBB4* with *CD133* and *FUT4* MBSCs markers in all human medulloblastoma cell lines demonstrating a positive correlation between *ERBB4* and both MBSC markers’ expression (Figure 5C), thereby linking high levels of *ERBB4* expression to MBSCs activity.

To directly explore the role of ERBB4 in the activity of MBSCs, we cultured DAOY and D283Med cells with *ERBB4* knock-down in the presence of stem cell medium. The results showed a marked decrease in the ability to form oncospheres, both in DAOY and D283Med cells (Figure 5D). Moreover, the ability to form secondary oncospheres was significantly impaired in *sh2* and *sh3* medulloblastoma cells (Figure S5C). In line with these results, *ERBB4* silencing significantly decreased the ability of colony formation (Figure 5E), suggesting that ERBB4 has a role in the maintenance of MBSCs.

In order to further characterize the impact of ERBB4 in MBSCs activity, we moved to in vivo experiments and injected limited dilution concentrations of knocked-down *ERBB4* DAOY and D283Med cells subcutaneously in immunocompromised mice. Strikingly, the frequency of tumor initiation was 1 in 475,710 and 946,994 in *sh2* and *sh3* cells, respectively, compared to 1 in 99,524 in the empty vector harboring DAOY cells (Figure 5F and Figure S5D) and 1 in 1,649,864 and infinite in *sh2* and *sh3* cells, respectively, compared to 1 in 441,838 in the empty vector harboring D283Med cells (Figure 5G and Figure S5D). The differences in stem cell frequencies between the tested groups were statistically significant, confirming that *ERBB4* inhibition limits tumor initiation and revealing an essential role for ERBB4 in MBSCs activity. 

### 3.6. ERBB4 Knock-Down Alters Multiple Processes and Pathways in Medulloblastoma Cells

**Figure 6:**
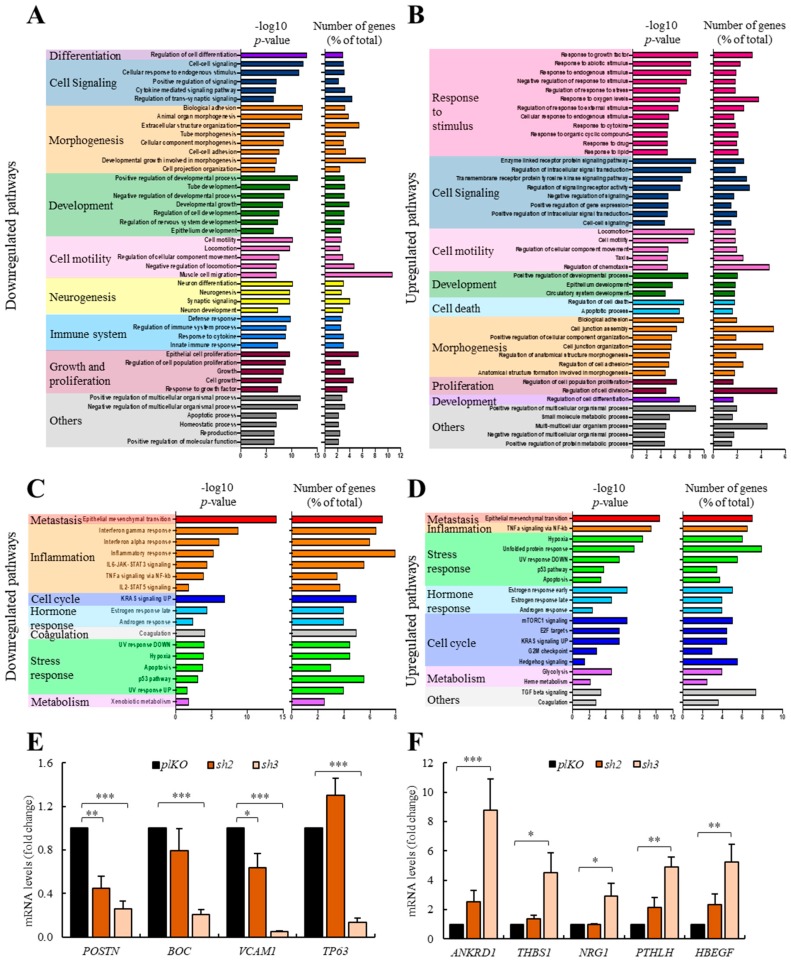
*ERBB4* knock-down alters multiple pathways of medulloblastoma cells. (**A**,**B**) Downregulated and upregulated pathways in *sh2* and *sh3* DAOY cells compared with *pLKO* cells when performing a GO gene sets biological processes analysis of Clariom S microarray results. (**C**,**D**) Downregulated and upregulated pathways in *sh2* and *sh3* DAOY cells compared with *pLKO* cells when performing a hallmark gene sets analysis of Clariom S microarray results. The *p*-value and the percentage of genes deregulated in each pathway are represented in all figures. (**E,F**) mRNA levels of downregulated and upregulated genes in control (*pLKO*) and *shERBB4* (*sh2* and *sh3*) DAOY cells (*n* ≥ 3). * *p* ≤ 0.05; ** *p* ≤ 0.01; and *** *p* ≤ 0.001

To investigate the pathways implicated when knocking-down *ERBB4* expression, we performed a transcriptomic analysis of *pLKO*, *sh2*, and *sh3* DAOY cells. We found 293 upregulated and 395 downregulated genes in *sh2* DAOY cells and 724 upregulated and 997 downregulated genes in *sh3* DAOY cells. All of these genes present a *p*-value lower than 0.05 and a fold change higher than 2. Then we found the common genes for both *shRNAs*, finding 179 upregulated and 297 downregulated genes (Table S1). These genes were further selected and performed a gene enrichment analysis with the GO gene sets, considering biological processes. The results obtained are consistent with our functional studies as processes such as cell motility, morphogenesis, development, cell growth and proliferation, and cell signaling were downregulated by knocking-down *ERBB4* (Figure 6A). On the contrary, cell death and response to stimulus were increased (Figure 6B). Among the identified genes, lower levels of stem cell markers such as *CD133* (prominin1), *DCLK1*, *LIN7*, *SOX4*, *SERPIN3*, or *TP63*; motility-related pathways such as *PRRX1*, *MMP7*, or *Claudin*; cell signaling as *FOS* or *JUN*; or cancer-related genes such as *K-RAS* were detected in *shERBB4* cells (Table S1). On the contrary, genes related to cell differentiation—*FGF5*; cell apoptosis—*APBB2*, *ANKRD1*, *PLAC8*; response to stress—*HA2FX*, *SERPINE1*, *CDH13*, *VEGFA*; and cell cycle and division—*CDK6*, *CDK15*, *CCND1*, *E2F7*, *CDC25a* were elevated in those cells (Table S1). We also performed a gene enrichment analysis based on hallmark gene sets. The results further showed alterations in cell cycle or stress response, finding a good correlation between both datasets, thus reinforcing the results obtained in the GO gene sets analysis, and revealed dysregulation in inflammation- or metabolism-related pathways (Figure 6C,D). Indeed, genes or inflammation markers such as *STAT3*, *OSMR*, *IL1R1*, *IL18R1*, *CXC16*, *IFITM1*, *SOCS2*, and *MGP* were decreased, whereas *IL1****α*** *IL7R* and *STAT5B* among others, or *PFKP* related to metabolism, were elevated in cells with *ERBB4* knock-down (Table S1). 

Next, we moved our attention to genes already related to medulloblastoma based on the literature and validated some of them, such as *POSTN* [52], *BOC* [53], *VCAM1* [54], and *TP63* [55] among the downregulated genes and *ANKRD1* [56], *THBS1* [57], *NRG1* [47], *PTHLH* [58], and *HBEGF* among the upregulated ones in DAOY and D283Med cells (Figure 6E,F and Figure S6A). Together, these analyses reveal advanced underlying molecular mechanisms of the activity of ERBB4 in medulloblastoma cells.

**Figure 7:**
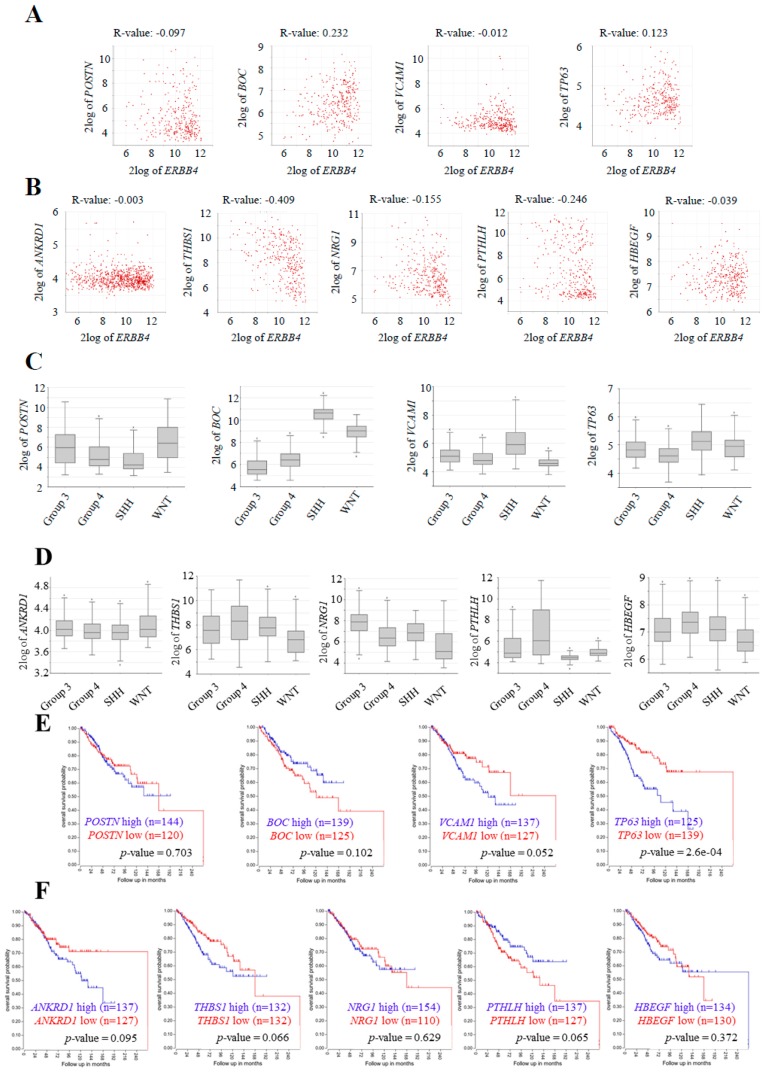
Expression and clinical impact of ERBB4 downstream genes in human samples of medulloblastoma. (**A**,**B**) Correlation analysis of (**A**) downregulated (*POSTN p*-value = 0.079, *BOC p*-value = 2.38 × 10^−5^, *VCAM1 p*-value = 0.835, and *TP63 p*-value = 0.027) and (**B**) upregulated (*ANKRD1 p*-value = 0.955, *THBS1 p*-value = 1.48 × 10^−14^, *NRG1 p*-value = 5.17 × 10^−3^, *PTHLH p*-value = 6.91-06, and *HBEGF p*-value = 0.482) with *ERBB4* expression for Cavalli cohort Group 4 patients’ (*n* = 326) data. Boxplot of the log2 of (**C**) downregulated and (**D**) upregulated genes in the indicated medulloblastoma subgroups in Cavalli cohort (*n* = 612). Kaplan–Meier curves for the Cavalli cohort Group 4 patients’ overall survival rates based on (**E**) downregulated and (**F**) upregulated expression (*p*-values are indicated in each graph). All results were obtained from hgserver1.

To further characterize the impact of the identified pathways and genes, we moved to clinical samples and completed a correlation study between ERBB4 expression and the validated genes in the Cavalli cohort, taking into account only Group 4 patients’ data. The results showed a statistically significant positive correlation of two of the genes (*BOC* and *TP63*) that appeared downregulated with ERBB4 knock-down (Figure 7A) and a negative correlation of all of the genes that appeared upregulated with ERBB4 knock-down; three of them (*THBS1*, *NRG1*, and *PTHLH*) showing statistically significance (Figure 7B). When performing the same analysis taking into account all patients’ data from the cohort, results were not as consistent (Figure S6B,C). These results reinforce the relation of these genes with ERBB4 expression, especially in the Group 4. 

Next, we analyzed their expression in the different subgroups using the information of all patients from the same cohort. We found that among the selected genes, three of the downregulated genes (*BOC*, *VCAM1*, and *TP63*) followed the same pattern as ERBB4 and showed a higher expression in Group 4 medulloblastomas (Figure 7C,D). Finally, we correlated their expression with patient outcome finding that altered *VCAM1*, *TP63*, *ANKRD1*, *THBS1*, and *PTHLH* levels correlated with lower patient survival in Group 4 medulloblastoma samples (Figure 7E,F). Moreover, when analyzing all medulloblastoma subgroups, elevated ANKRD1 and NRG1 expression correlated with lower patient survival (Figure S7A,B). All these results extrapolate the findings in vitro to clinical samples in vivo, and reveal several promising genes and pathways related to ERBB4 activity in medulloblastoma progression.

## 4. Discussion

The cerebellum development is a postnatal process, which is tightly regulated by the expression of several genes [59]. When a dysregulation of this process occurs, medulloblastoma can be generated [4]. Several genes and signaling pathways that direct embryonic developmental processes are also implicated in tumor formation and progression, such as WNT and SHH pathways, and their dysregulation is linked to medulloblastoma formation [60,61]. ERBB4 is a tyrosine kinase membrane receptor that has been described to be essential for the normal neural development and maintenance of nervous system in part controlling cell migration [62,63,64]. Indeed, its genetic deletion leads to neurodevelopmental impairment affecting neuronal migration in multiple brain structures from the cortex to the cerebellum [35]. 

In our study, we first investigated the expression of ERBB4 and its ligands NRG1 and HBEGF in mice cerebella. These analyses indicate that ERBB4 and its active form, P-ERBB4, are expressed since early stages of embryonic development, especially in the EGL of the developing cerebellum, the proliferative layer of the cerebellum. When ERBB4 ligands were analyzed, both were detected at mRNA level and presented different patterns of expression. At protein level, we were not able to detect NRG1 expression whilst we found expression of HBEGF [65]. Interestingly, we show that this ligand expression increases during cerebellar development in vivo, reaching the higher levels at p18, when cerebellum is totally developed. Surprisingly, ERBB4 and HBEGF ligand expression do not colocalize, since we detected HBEGF expression in the IGL of the cerebellum. The results obtained when treating CGNPs with HBEGF and NRG1 ligands reveal higher activation of ERBB4 when treating cells with NRG1, showing that even if its expression in vivo is low, this ligand may have a role in the activation and function of ERBB4. Further investigation will be needed to understand the interactions of ERBB4 with these ligands in vivo during development. Our results in *ErbB4* mutant mice show an aberrant development of the cerebellum with ectopic differentiated cell clusters in the EGL and misaligned GFAP-positive radial fibers. We also found aberrant migration of CGNPs in *ErbB4* mutant mice cerebella, in agreement with previous results [35], and also in medulloblastoma tumor cells. Moreover, our transcriptomic results revealed several novel genes related to morphogenesis, development, and cell migration, thus demonstrating the essential role of ERBB4 in the migratory capacity of cells and in the formation of the cerebellar structures. 

Our data confirms that ERBB4 is also relevant for protecting CGNPs and medulloblastoma tumor cells from apoptosis in vitro and in vivo. Thus, *ErbB4 KO* mice CGNPs treated with an apoptotic drug (dexamethasone) and hNRG1, as an ERBB4 activator, were are sensitive to apoptosis than controls. Moreover, *ErbB4* deletion in the medulloblastoma-prone *SmoM2* mice also promotes apoptosis activation. In line with these results, experimental silencing of *ERBB4* in medulloblastoma cells significantly impairs cell viability and tumor progression as a consequence of apoptosis induction in vitro and in vivo, respectively. In line with these results, it has been described that ERBB4 protects ventricular myocytes [66] and neurons [67] from apoptosis and, indeed, our transcriptomic results revealed genes related to apoptosis and cell death increased in *ERBB4* knocked-down cells.

Moreover, our data revealed that *ERBB4* expression is enriched in the MBSCs population, and it is important for their activity since *ERBB4* knocked-down cells present a decreased oncosphere and foci-formation and self-renewal capacity in vitro, and tumor initiation in vivo. These results demonstrate that high levels of *ERBB4* are involved in maintaining the MBSC population. In agreement with this idea, *ERBB4* has been related to stem cell function during embryonic developmental processes [30] and in human colon cancer [68]. In summary, our results indicate that ERBB4 expression is necessary for MBSC maintenance, likely regulating the interplay between self-renewal and differentiation. In support of this idea, transcriptomic analysis showed that multiple pathways and genes related to stem cell activity are altered in *ERBB4* knocked-down cells. Moreover, this analysis revealed an upregulation in response to stimulus and cell death. These last findings might be considered as advantageous for the consideration of a therapy against ERBB4 receptor, since an upregulation of response to stimulus, such radiotherapy or chemotherapy, may reduce the resistance to these therapies, and activation of cell death could help to reduce or limit tumor size.

Translating these pre-clinical results to the clinic, *ERBB4* expression is elevated in medulloblastoma samples where its high levels are associated with poor patient survival. These data confirm the prognostic significance of *ERBB4* expression and, to our knowledge, provide the first evidence of *ERBB4* expression as a negative prognostic biomarker in medulloblastoma. When ERBB4 expression was analyzed in the different groups, its levels were higher in Group 4 medulloblastomas in all studied cohorts. These results are in agreement with a previous study [69]. Interestingly, *ERBB4* expression was not only a Group 4 biomarker, but also a prognostic biomarker in this subset of patients, since high *ERBB4* expression correlated poorly with patient survival in this group of patients. Finally, we identified and validated a set of 9 genes as downstream targets of *ERBB4* activity in medulloblastoma. Among them, NRG1 and HBEGF ligands were elevated in *ERBB4* knocked-down cells. *TP63* and *PTHLH* genes seem to deserve special interest, since their expression correlates positively and negatively with *ERBB4* in medulloblastoma cells and samples, respectively, and their expression is related to patient prognosis, following the same pattern as *ERBB4*. *TP63* is an important transcriptor factorthat belongs to the TP53 family, and is a key developmental and stem cell factor with diverse and complex functions in tumorigenesis. Previous studies linking TP63 to the NRG1 and STAT family supports the association identified in this work [55]. *PTHLH* (parathyroid hormone related protein) is an important growth factor that exhibits pleiotropic effects, including tumor cell growth, death, and differentiation, which has been linked to the SHH pathway [58]. 

In summary, our work shows that *ERBB4* expression is highly enriched in the pool of MBSCs, and its inactivation significantly impairs their malignant properties of proliferation and tumor initiation and progression. Moreover, we reveal that *ERBB4* is overexpressed in Group 4 medulloblastomas, the most common group, and that high expression levels of this receptor are associated with shorter overall patient survival. Based on our results, we also postulate that ERBB4 has an important role in cerebellum development, governing and maintaining CGNPs’ proliferation and migration. Taken together, our data depict a previously unknown role for ERBB4 as a central player of medulloblastoma biology, prognosis, and therapy, as well as reveal underlying molecular pathways for ERBB4 activity. 

## 5. Conclusions 

Our results show that ERBB4 plays a critical role for stem cell population in homeostasis during cerebellum development and also in pathological conditions in medulloblastoma. Multiple processes and molecular pathways seem to be modulated by ERBB4, being apoptosis, migration and self-renewal/differentiation the most important ones. Finally, we firmly established that *ERBB4* behaves as an oncogene in medulloblastoma.

## Supplementary Materials

The following are available online at https://www.mdpi.com/2072-6694/12/4/997/s1, Figure S1: Western Blot analysis of the indicated proteins in cerebellum from SmoM2;ErbB4 Het and KO p17 mice, Figure S2: *p*-values of the differences of ERBB4 expression in the indicated medulloblastoma subgroups in (A) Northcott PA et al. 2012 cohort (*n* = 1087), (B) Robinson G et al. 2012 cohort (*n* = 76), (C) Remke M et al. 2011 cohort (*n* = 64) and (D) Cavalli FMG et al. 2017 cohort (*n* = 612), Figure S3: (A) plKO, sh2 and sh3 DAOY and D283Med cells number at day 1, 3 and 5 after seeding (*n* ≥ 3). (B) Representative images and quantification of phospho-Histone-3 (PH3) positive cells in sh2 and sh3 relative to plKO DAOY cells (*n* ≥ 3). (C) Cell cycle assay measuring the number of cells in each cell cycle phase in plKO, sh2 and sh3 DAOY cells (*n* ≥ 3). (D) Representative immunofluorescence images of cleaved-Caspase-3 (cC3) positive cells in plKO, sh2 and sh3 DAOY and D283Med cells (*n* ≥ 3). (E) Representative images and quantification of the migration assay (*n* ≥ 3), Figure S4: (A) Quantification of weight of tumors generated after subcutaneous injection of DAOY and D283Med pLKO and sh2 cells (*n* = 8). (B) Representative images of the immunohistochemical staining of cC3 and KI67 in tumors generated after subcutaneous injection of DAOY pLKO and sh2 cells (*n* ≥ 3), Figure S5: (A) mRNA levels of the indicated stem cell markers of cells grown in serum (D283Med or D341Med) or in stem cell media as oncospheres (spheres) (*n* ≥ 3). (B) Western Blot analysis of the indicated proteins in protein extracts of cells grown in serum (D283Med or D341Med) or in stem cell media as oncospheres (spheres) (*n* ≥ 3). (C) Quantification of the number of 1ry and 2ry oncospheres formed from plKO, sh2 and sh3 DAOY cells (*n* ≥ 3). (D) Number of stem cells needed to initiate a tumor in the indicated conditions with DAOY and D283Med cells, Figure S6: (A) mRNA levels of downregulated and upregulated genes identified in the microarray conducted in Figure 6 in plKO and sh3 D283Med cells (*n* ≥ 3). (B,C) Correlation analysis of (B) downregulated (POSTN *p*-value = 2.91 × 10^−3^, BOC *p*-value = 2.08 × 10^−33^, VCAM1 *p*-value = 0.121 and TP63 *p*-value = 9.54 × 10^−7^) and (C) upregulated (ANKRD1 *p*-value = 0.013, THBS1 *p*-value = 6.78 × 10^−3^, NRG1 *p*-value = 1.07 × 10^−5^, PTHLH *p*-value = 7.95 × 10^−6^ and HBEGF *p*-value = 3.95 × 10^−8^) genes with ERBB4 expression for Cavalli FMG et al. 2017 cohort’s all patients’ (*n* = 612) data, Figure S7: (A,B) Kaplan–Meier curves for the Cavalli FMG et al. 2017 cohort’s all patients’ overall survival rates based on (A) downregulated and (B) upregulated genes (*p*-values are indicated in each graph). All results are obtained from hgserver1, Figure S8: The uncropped blots and molecular weight markers of Figure 2B, Figure S9: The uncropped blots and molecular weight markers of Figure 2C, Figure S10: The uncropped blots and molecular weight markers of Figure 2D, Figure S11: The uncropped blots and molecular weight markers of Figure 4A, Table S1: Aldaregia List of genes.
