# Supplementary material for: Erbb4 Is Required for Cerebellar Development and Malignant Phenotype of Medulloblastoma
Source: Cancers (Basel). 2020 Apr 17;12(4):997. doi: 10.3390/cancers12040997 (PMC7226104; doi:10.3390/cancers12040997)
Supplement: Supplementary file 1 [file cancers-12-00997-s001.zip › cancers-774606-suppl/1 Aldaregia et al Figure Suppl Resubmision final.pptx]

## Slide 1
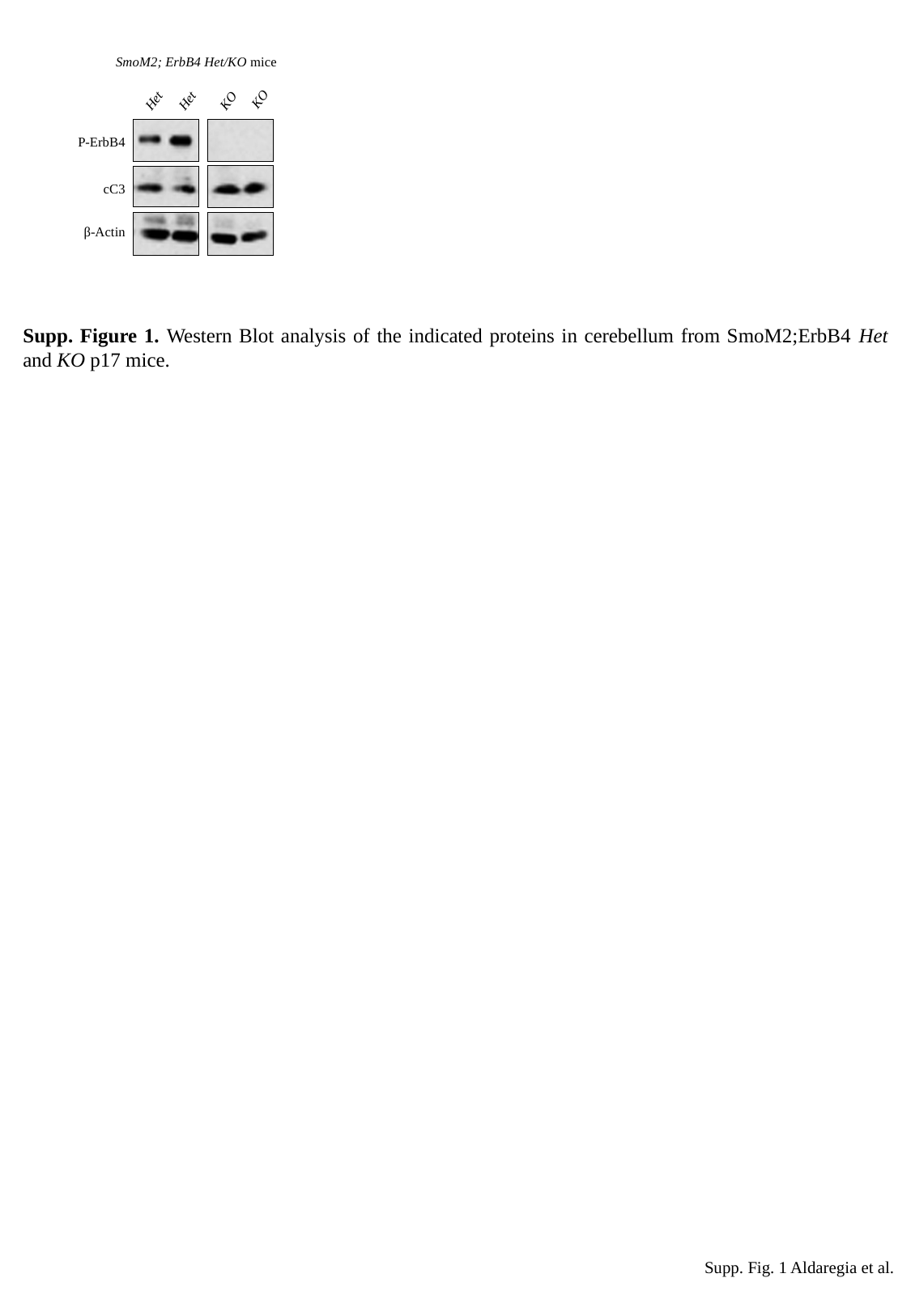

SmoM2; ErbB4 Het/KO mice
KO
Het
Het
KO
P-ErbB4
cC3
β-Actin
Supp. Figure 1. Western Blot analysis of the indicated proteins in cerebellum from SmoM2;ErbB4 Het and KO p17 mice.
Supp. Fig. 1 Aldaregia et al.

## Slide 2
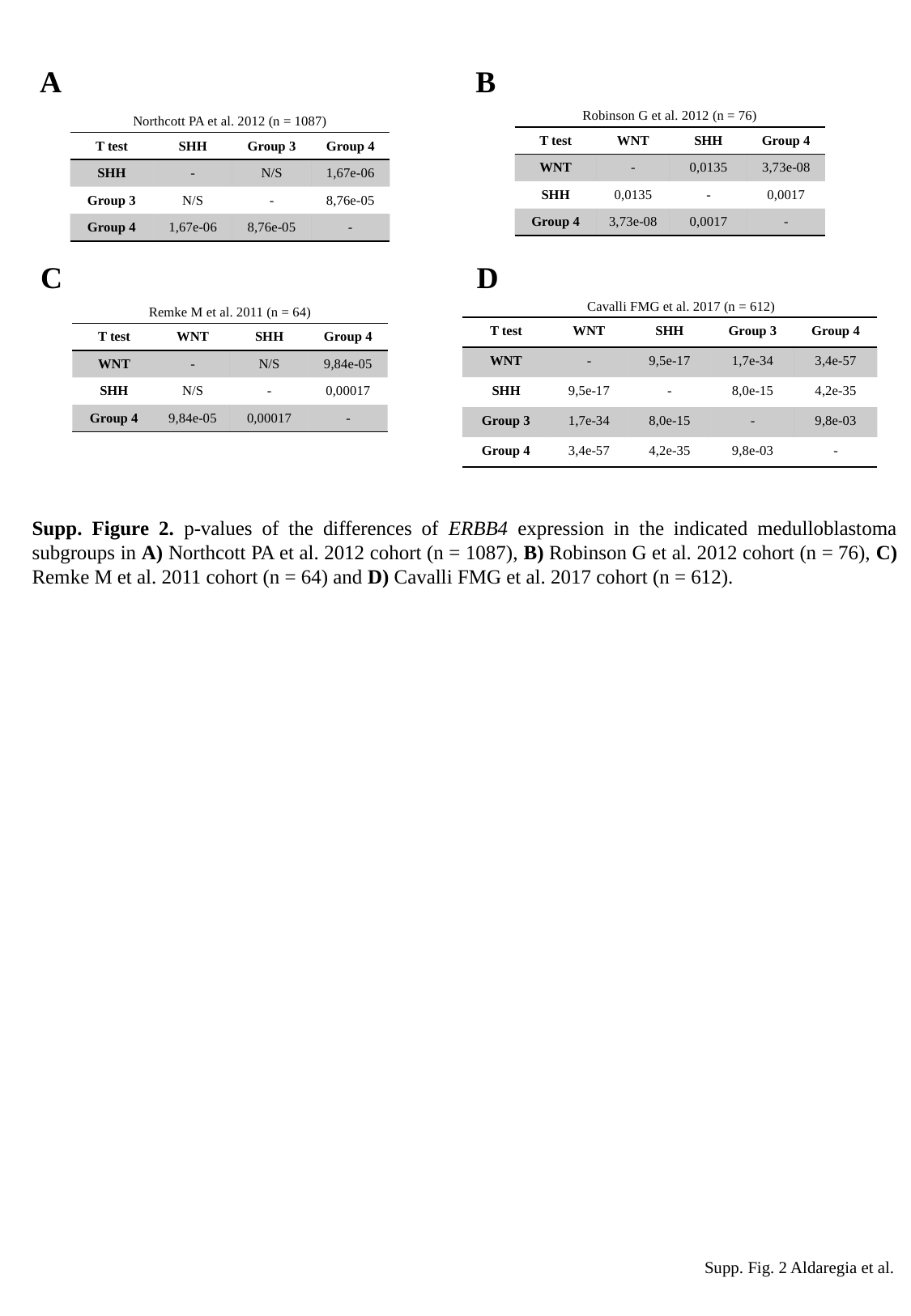

A
B
Robinson G et al. 2012 (n = 76)
Northcott PA et al. 2012 (n = 1087)
| T test | WNT | SHH | Group 4 |
| --- | --- | --- | --- |
| WNT | - | 0,0135 | 3,73e-08 |
| SHH | 0,0135 | - | 0,0017 |
| Group 4 | 3,73e-08 | 0,0017 | - |
| T test | SHH | Group 3 | Group 4 |
| --- | --- | --- | --- |
| SHH | - | N/S | 1,67e-06 |
| Group 3 | N/S | - | 8,76e-05 |
| Group 4 | 1,67e-06 | 8,76e-05 | - |
C
D
Cavalli FMG et al. 2017 (n = 612)
Remke M et al. 2011 (n = 64)
| T test | WNT | SHH | Group 3 | Group 4 |
| --- | --- | --- | --- | --- |
| WNT | - | 9,5e-17 | 1,7e-34 | 3,4e-57 |
| SHH | 9,5e-17 | - | 8,0e-15 | 4,2e-35 |
| Group 3 | 1,7e-34 | 8,0e-15 | - | 9,8e-03 |
| Group 4 | 3,4e-57 | 4,2e-35 | 9,8e-03 | - |
| T test | WNT | SHH | Group 4 |
| --- | --- | --- | --- |
| WNT | - | N/S | 9,84e-05 |
| SHH | N/S | - | 0,00017 |
| Group 4 | 9,84e-05 | 0,00017 | - |
Supp. Figure 2. p-values of the differences of ERBB4 expression in the indicated medulloblastoma subgroups in A) Northcott PA et al. 2012 cohort (n = 1087), B) Robinson G et al. 2012 cohort (n = 76), C) Remke M et al. 2011 cohort (n = 64) and D) Cavalli FMG et al. 2017 cohort (n = 612).
Supp. Fig. 2 Aldaregia et al.

## Slide 3
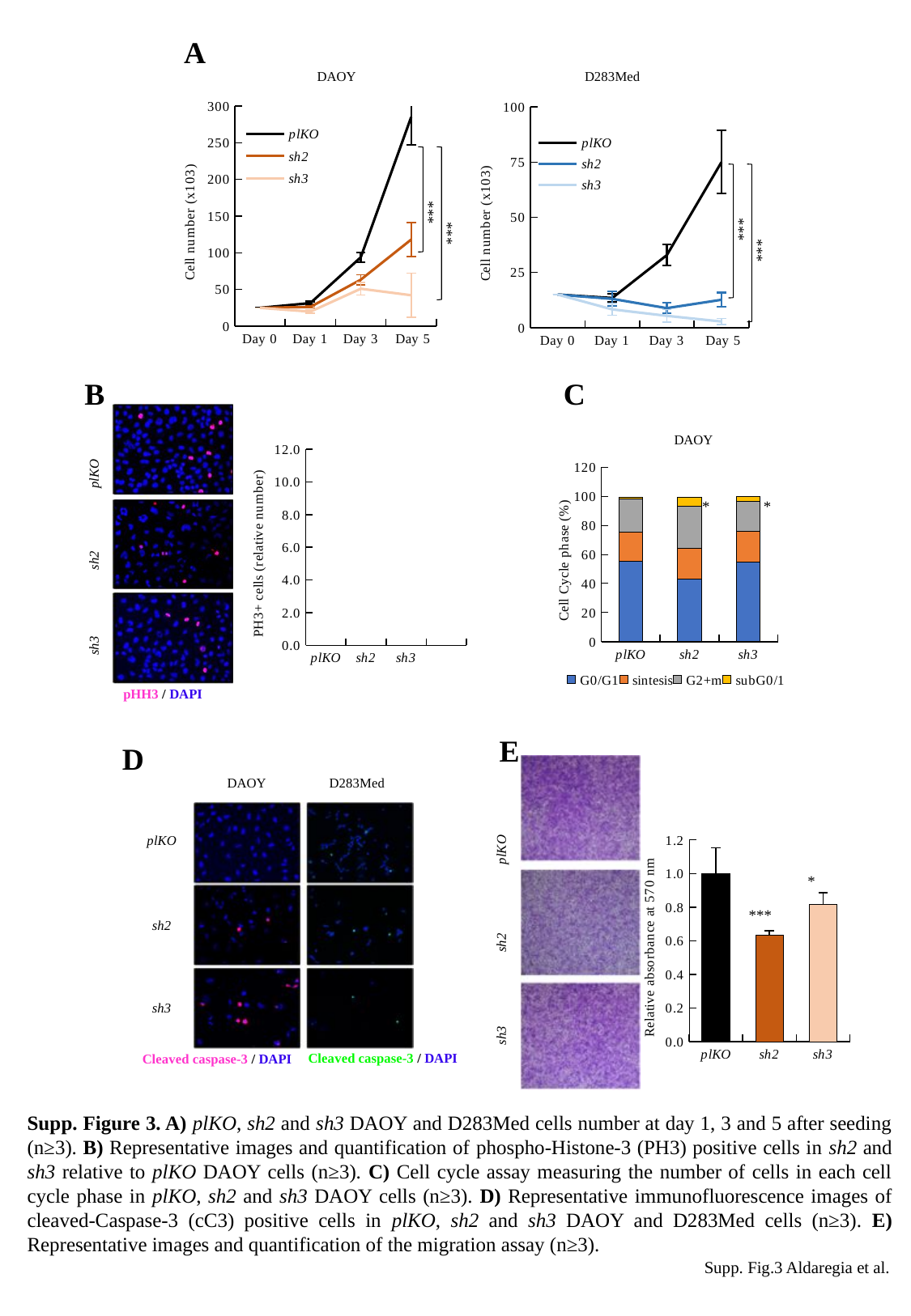

A
DAOY
D283Med
### Chart
| Category | plKO | sh2 | sh3 |
|---|---|---|---|
| Day 0 | 25.0 | 25.0 | 25.0 |
| Day 1 | 31.5 | 26.3 | 19.83333333333328 |
| Day 3 | 93.87499999999999 | 63.3 | 51.04166666666646 |
| Day 5 | 284.9444444444445 | 118.05 | 42.16666666666651 |
### Chart
| Category | plKO | sh2 | sh3 |
|---|---|---|---|
| Day 0 | 15.0 | 15.0 | 15.0 |
| Day 1 | 13.43333333333333 | 13.04861111111111 | 8.275 |
| Day 3 | 32.86666666666648 | 8.833333333333336 | 5.362499999999992 |
| Day 5 | 75.0 | 12.63333333333333 | 2.786111111111112 |
***
***
***
***
B
C
DAOY
### Chart
| Category | |
|---|---|
| plKO | 1.0 |
| sh2 | 1.284017298428407 |
| sh3 | 0.862035693953238 |
### Chart
| Category | G0/G1 | sintesis | G2+m | subG0/1 |
|---|---|---|---|---|
| plKO | 55.1375 | 20.2375 | 22.6125 | 1.4375 |
| sh2 | 43.1125 | 20.9 | 28.975 | 6.324999999999979 |
| sh3 | 54.95 | 20.9125 | 20.425 | 3.45 |*
*
plKO
sh2
sh3
pHH3 / DAPI
E
D
D283Med
DAOY
plKO
### Chart
| Category | |
|---|---|
| plKO | 1.0 |
| sh2 | 0.631793979127751 |
| sh3 | 0.816374546075473 |plKO
*
***
sh2
sh2
sh3
sh3
Cleaved caspase-3 / DAPI
Cleaved caspase-3 / DAPI
Supp. Figure 3. A) plKO, sh2 and sh3 DAOY and D283Med cells number at day 1, 3 and 5 after seeding (n≥3). B) Representative images and quantification of phospho-Histone-3 (PH3) positive cells in sh2 and sh3 relative to plKO DAOY cells (n≥3). C) Cell cycle assay measuring the number of cells in each cell cycle phase in plKO, sh2 and sh3 DAOY cells (n≥3). D) Representative immunofluorescence images of cleaved-Caspase-3 (cC3) positive cells in plKO, sh2 and sh3 DAOY and D283Med cells (n≥3). E) Representative images and quantification of the migration assay (n≥3).
Supp. Fig.3 Aldaregia et al.

## Slide 4
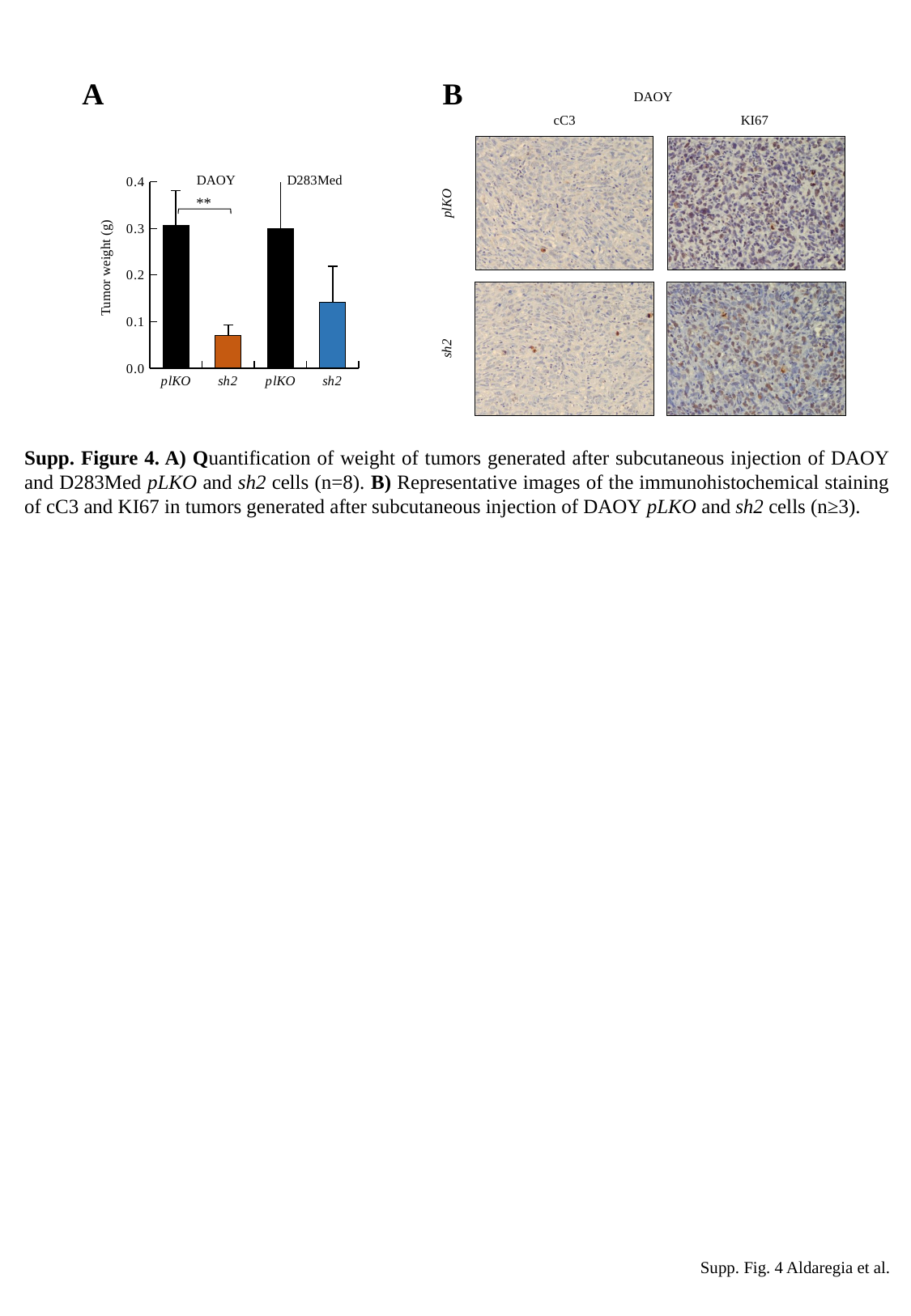

A
B
DAOY
cC3
KI67
D283Med
DAOY
### Chart
| Category | |
|---|---|
| plKO | 0.3065 |
| sh2 | 0.06925 |
| plKO | 0.298714285714286 |
| sh2 | 0.140625 |
**
plKO
Tumor weight (g)
sh2
Supp. Figure 4. A) Quantification of weight of tumors generated after subcutaneous injection of DAOY and D283Med pLKO and sh2 cells (n=8). B) Representative images of the immunohistochemical staining of cC3 and KI67 in tumors generated after subcutaneous injection of DAOY pLKO and sh2 cells (n≥3).
Supp. Fig. 4 Aldaregia et al.

## Slide 5
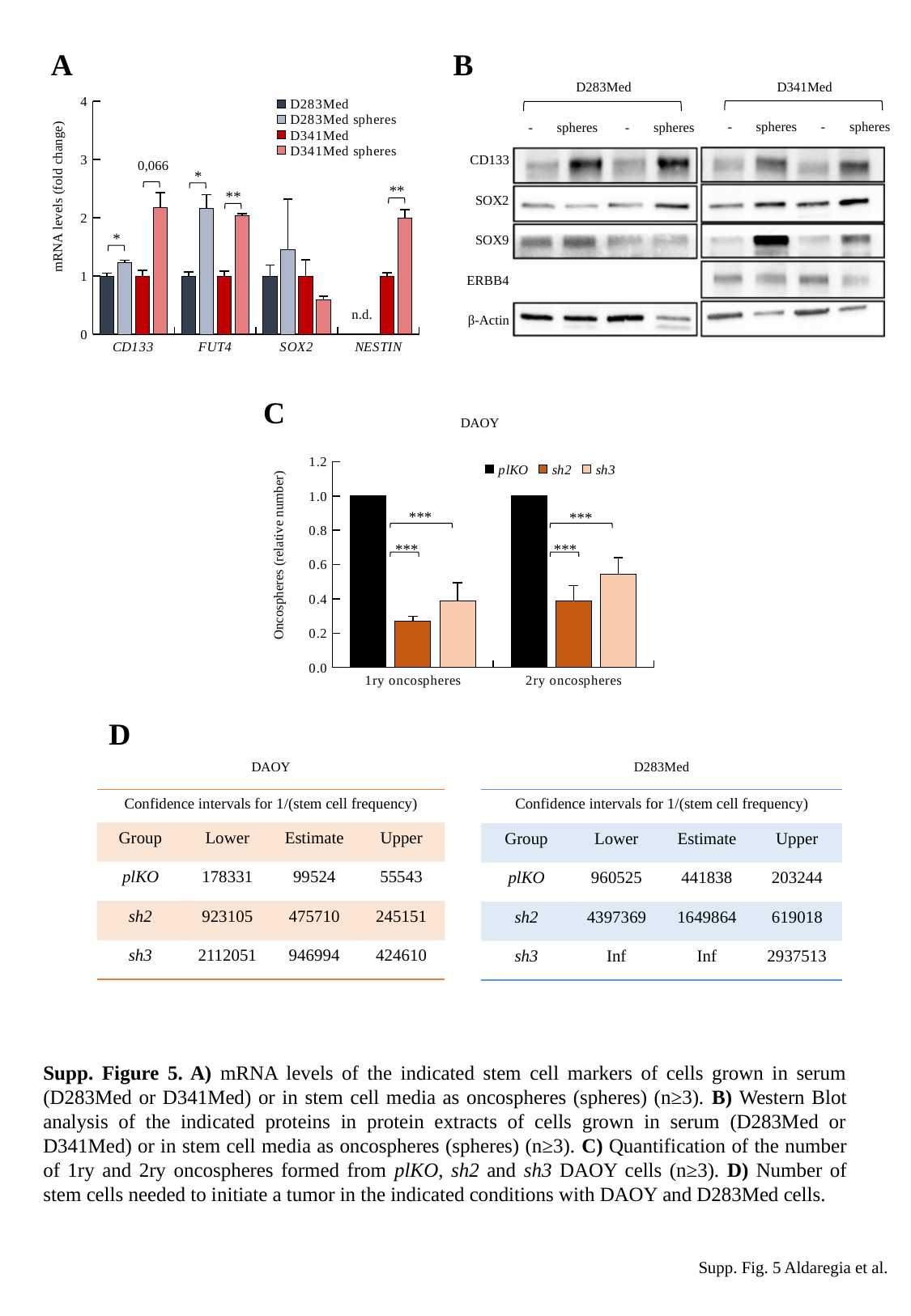

A
B
D341Med
D283Med
### Chart
| Category | D283Med | D283Med spheres | D341Med | D341Med spheres |
|---|---|---|---|---|
| CD133 | 1.0 | 1.236485442316545 | 1.0 | 2.17372465647677 |
| FUT4 | 1.0 | 2.167638642511135 | 1.0 | 2.029835848411616 |
| SOX2 | 1.0 | 1.452489745614511 | 1.0 | 0.587062976511051 |
| NESTIN | None | None | 1.0 | 2.000427329166904 | - spheres - spheres
 - spheres - spheres
CD133
0,066
*
**
mRNA levels (fold change)
**
SOX2
*
SOX9
ERBB4
n.d.
β-Actin
C
DAOY
### Chart
| Category | plKO | sh2 | sh3 |
|---|---|---|---|
| 1ry oncospheres | 1.0 | 0.268148200367348 | 0.386815583594934 |
| 2ry oncospheres | 1.0 | 0.387556240935607 | 0.542661857950943 |
***
***
***
***
Oncospheres (relative number)
D
DAOY
D283Med
| Confidence intervals for 1/(stem cell frequency) | | | |
| --- | --- | --- | --- |
| Group | Lower | Estimate | Upper |
| plKO | 178331 | 99524 | 55543 |
| sh2 | 923105 | 475710 | 245151 |
| sh3 | 2112051 | 946994 | 424610 |
| Confidence intervals for 1/(stem cell frequency) | | | |
| --- | --- | --- | --- |
| Group | Lower | Estimate | Upper |
| plKO | 960525 | 441838 | 203244 |
| sh2 | 4397369 | 1649864 | 619018 |
| sh3 | Inf | Inf | 2937513 |
Supp. Figure 5. A) mRNA levels of the indicated stem cell markers of cells grown in serum (D283Med or D341Med) or in stem cell media as oncospheres (spheres) (n≥3). B) Western Blot analysis of the indicated proteins in protein extracts of cells grown in serum (D283Med or D341Med) or in stem cell media as oncospheres (spheres) (n≥3). C) Quantification of the number of 1ry and 2ry oncospheres formed from plKO, sh2 and sh3 DAOY cells (n≥3). D) Number of stem cells needed to initiate a tumor in the indicated conditions with DAOY and D283Med cells.
Supp. Fig. 5 Aldaregia et al.

## Slide 6
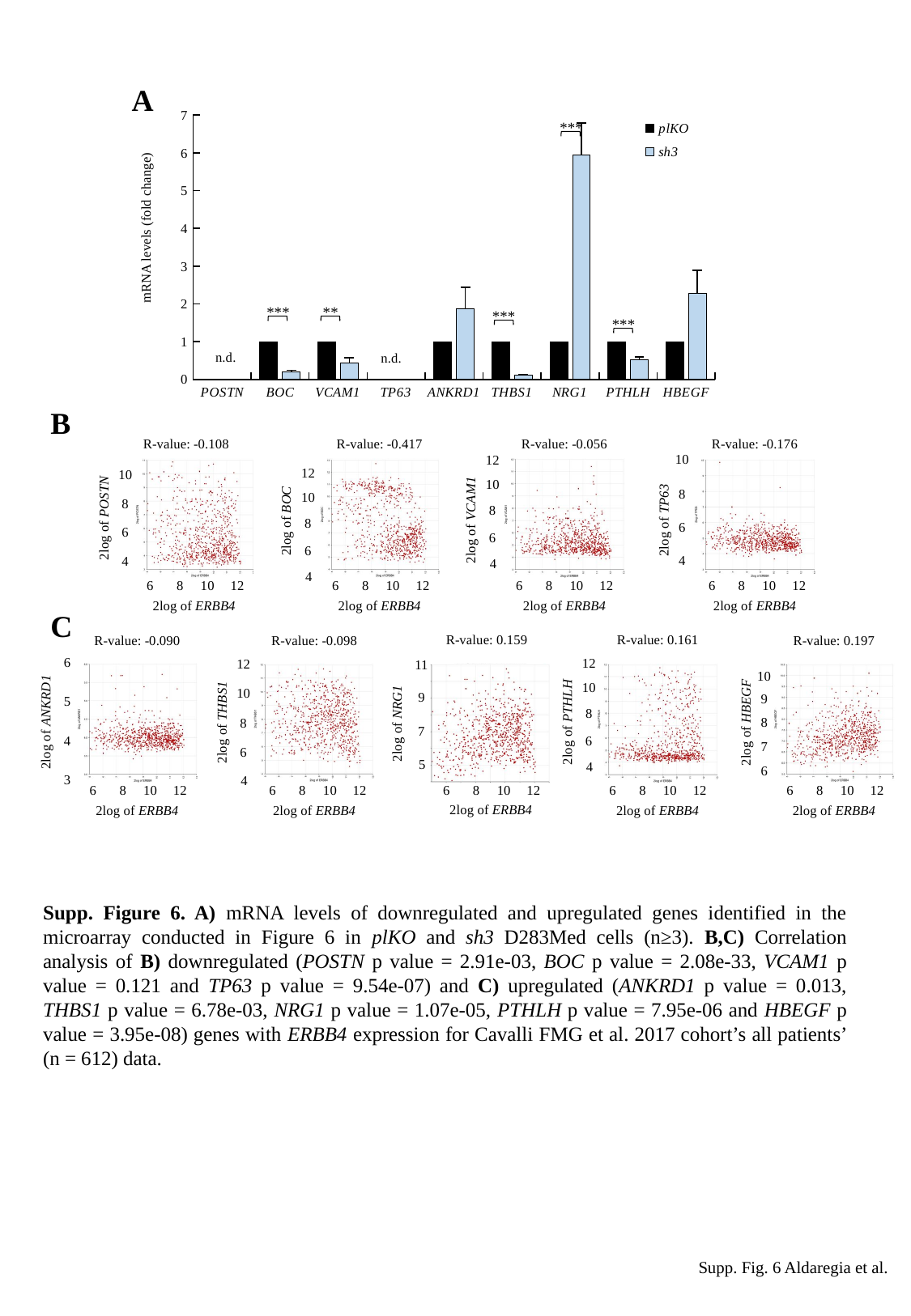

A
### Chart
| Category | plKO | sh3 |
|---|---|---|
| POSTN | None | None |
| BOC | 1.0 | 0.200368827787404 |
| VCAM1 | 1.0 | 0.430017857112884 |
| TP63 | None | None |
| ANKRD1 | 1.0 | 1.877295362688312 |
| THBS1 | 1.0 | 0.121483781220018 |
| NRG1 | 1.0 | 5.946258749411192 |
| PTHLH | 1.0 | 0.518610961554185 |
| HBEGF | 1.0 | 2.273337215509246 |***
mRNA levels (fold change)
***
**
***
***
n.d.
n.d.
B
R-value: -0.108
R-value: -0.417
R-value: -0.056
R-value: -0.176
10
12
12
10
10
8
10
8
8
2log of POSTN
2log of VCAM1
2log of TP63
2log of BOC
8
6
6
6
6
4
4
4
4
6
8
10
12
6
8
10
12
6
8
10
12
6
8
10
12
2log of ERBB4
2log of ERBB4
2log of ERBB4
2log of ERBB4
C
R-value: 0.159
R-value: 0.161
R-value: -0.098
R-value: -0.090
R-value: 0.197
6
12
12
11
10
10
10
9
9
5
8
2log of THBS1
2log of PTHLH
2log of HBEGF
2log of ANKRD1
8
8
2log of NRG1
7
6
4
7
6
5
4
6
3
4
6
8
10
12
6
8
10
12
6
8
10
12
6
8
10
12
6
8
10
12
2log of ERBB4
2log of ERBB4
2log of ERBB4
2log of ERBB4
2log of ERBB4
Supp. Figure 6. A) mRNA levels of downregulated and upregulated genes identified in the microarray conducted in Figure 6 in plKO and sh3 D283Med cells (n≥3). B,C) Correlation analysis of B) downregulated (POSTN p value = 2.91e-03, BOC p value = 2.08e-33, VCAM1 p value = 0.121 and TP63 p value = 9.54e-07) and C) upregulated (ANKRD1 p value = 0.013, THBS1 p value = 6.78e-03, NRG1 p value = 1.07e-05, PTHLH p value = 7.95e-06 and HBEGF p value = 3.95e-08) genes with ERBB4 expression for Cavalli FMG et al. 2017 cohort’s all patients’ (n = 612) data.
Supp. Fig. 6 Aldaregia et al.

## Slide 7
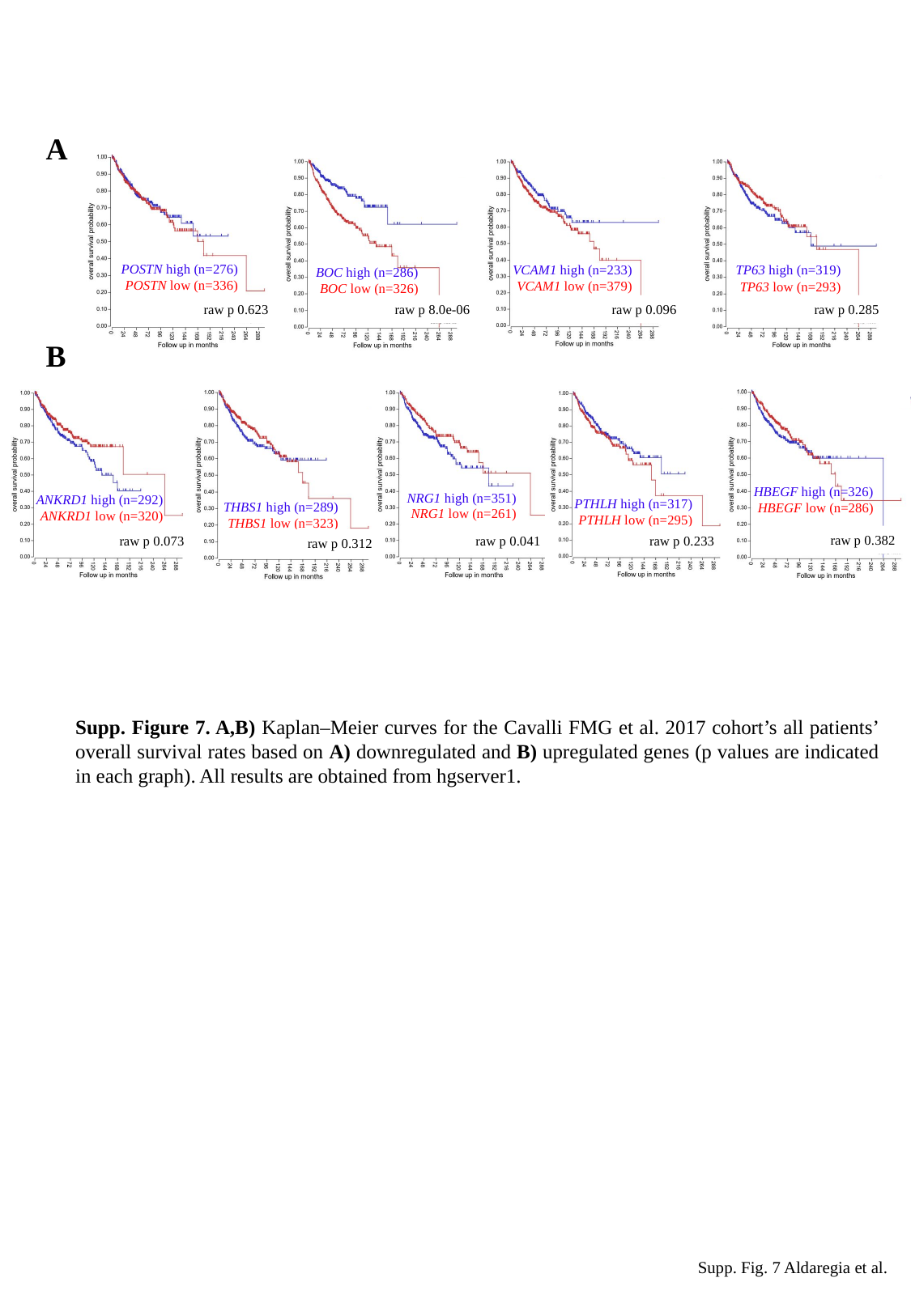

A
POSTN high (n=276)
POSTN low (n=336)
VCAM1 high (n=233)
VCAM1 low (n=379)
TP63 high (n=319)
TP63 low (n=293)
BOC high (n=286)
BOC low (n=326)
raw p 0.623
raw p 8.0e-06
raw p 0.096
raw p 0.285
B
HBEGF high (n=326)
HBEGF low (n=286)
NRG1 high (n=351)
NRG1 low (n=261)
ANKRD1 high (n=292)
ANKRD1 low (n=320)
PTHLH high (n=317)
PTHLH low (n=295)
THBS1 high (n=289)
THBS1 low (n=323)
raw p 0.382
raw p 0.233
raw p 0.073
raw p 0.041
raw p 0.312
Supp. Figure 7. A,B) Kaplan–Meier curves for the Cavalli FMG et al. 2017 cohort’s all patients’ overall survival rates based on A) downregulated and B) upregulated genes (p values are indicated in each graph). All results are obtained from hgserver1.
Supp. Fig. 7 Aldaregia et al.
