# Supplementary figures and images for: Erbb4 Is Required for Cerebellar Development and Malignant Phenotype of Medulloblastoma
Source: Cancers (Basel). 2020 Apr 17;12(4):997. doi: 10.3390/cancers12040997 (PMC7226104; doi:10.3390/cancers12040997)

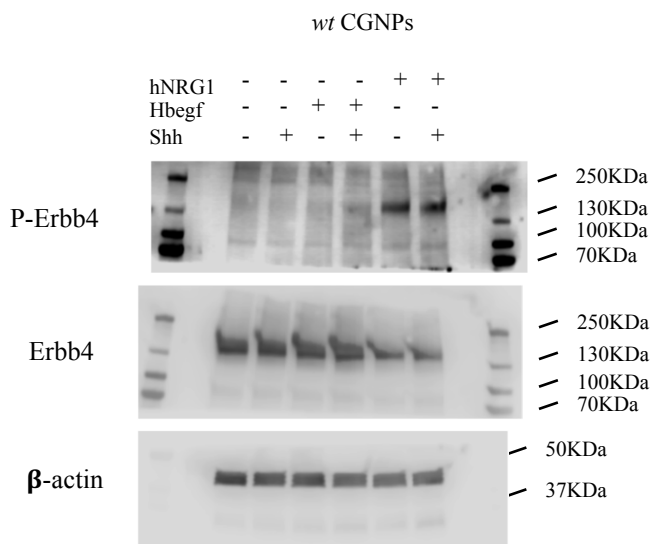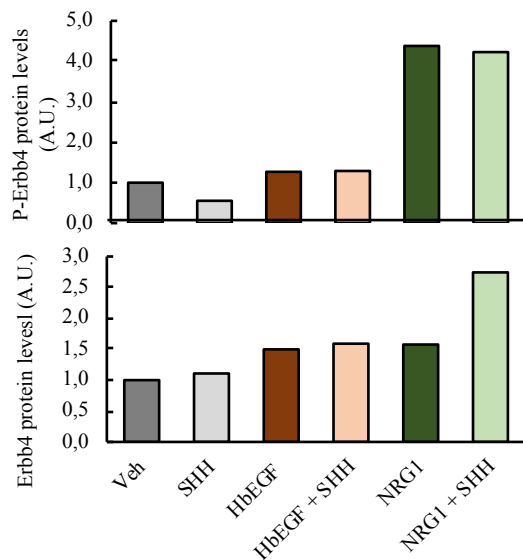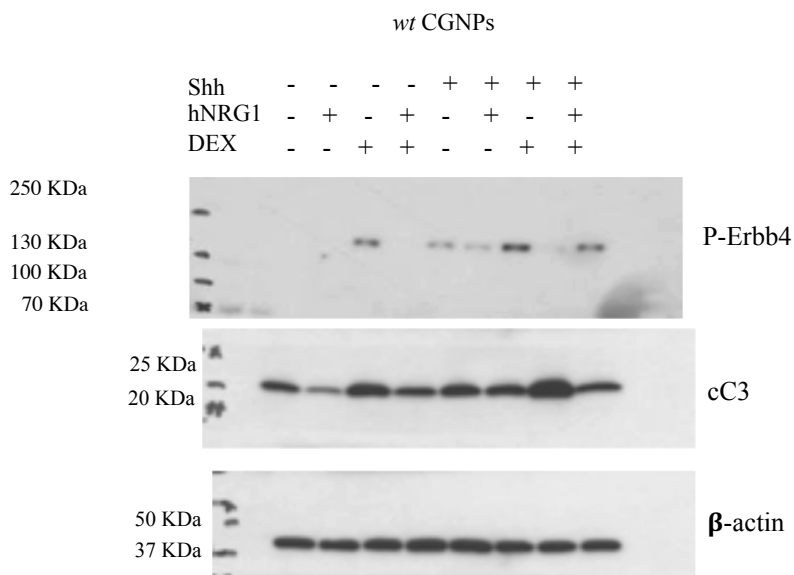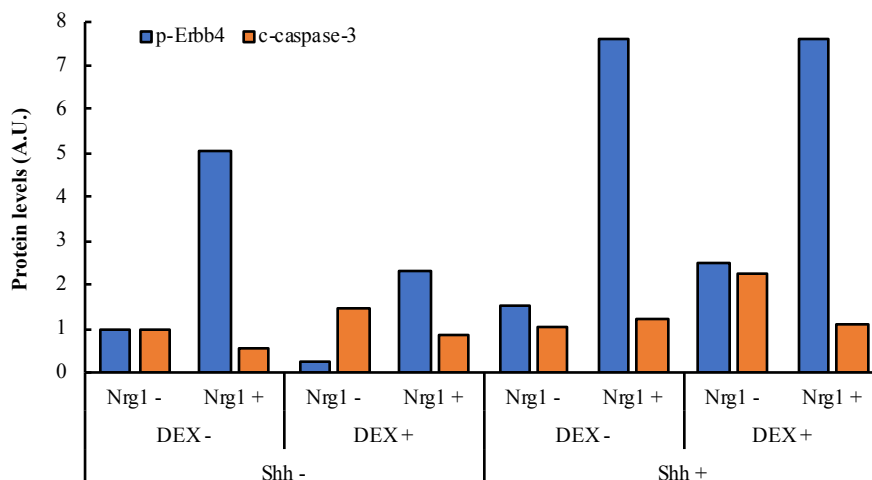

ERBB4 KO  
CGNPs

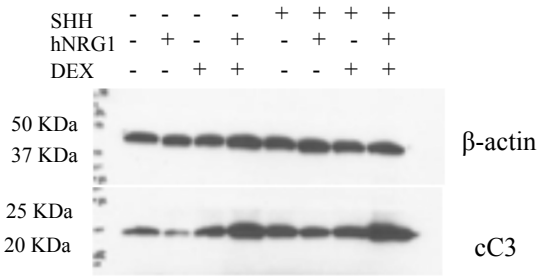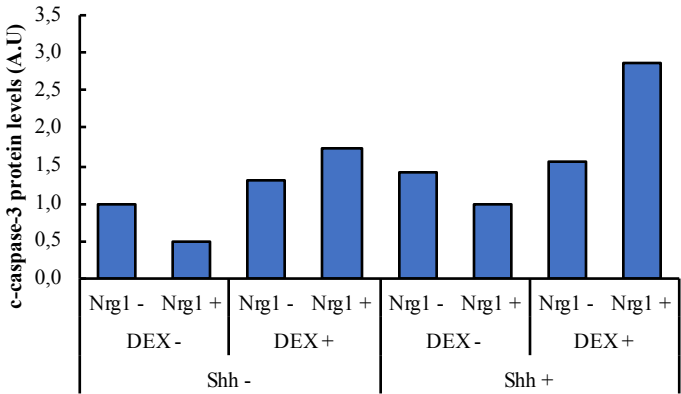

p15

ErbB4Het/KO + SmoM2 tumors

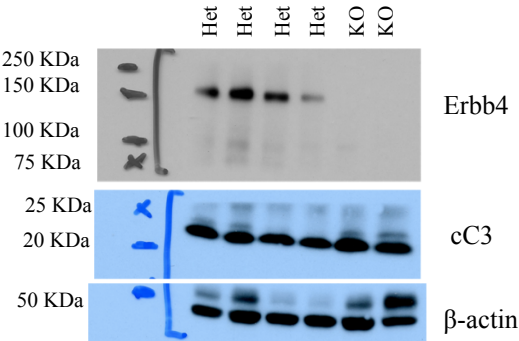

p17

ErbB4Het/KO + SmoM2 tumors

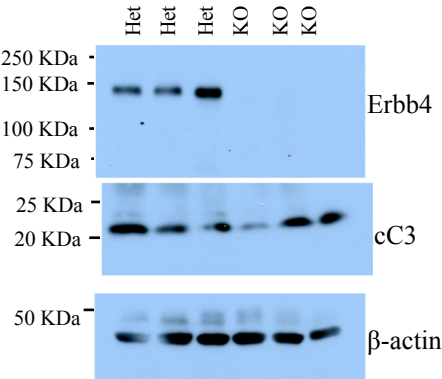

Supplement: Supplementary file 1 [file cancers-12-00997-s001.zip › cancers-774606-suppl/cancers-774606-website blot.pdf]
